# Can natural products stop the SARS-CoV-2 virus? A docking and molecular dynamics study of a natural product database

**DOI:** 10.4155/fmc-2020-0248

**Published:** 2021-01-08

**Authors:** Jurica Novak, Hrvoje Rimac, Shivananda Kandagalla, Maria A Grishina, Vladimir A Potemkin

**Affiliations:** ^1^Higher Medical & Biological School, Laboratory of Computational Modeling of Drugs, South Ural State University, 20-A, Tchaikovsky Str., Chelyabinsk 454080, Russia; ^2^Department of Medicinal Chemistry, University of Zagreb, Faculty of Pharmacy & Biochemistry, Ante Kovacica 1, 10000 Zagreb, Croatia

**Keywords:** 3CLpro, COVID-19, futalosine, molecular dynamics, natural product atlas, SARS-CoV-2

## Abstract

**Background:** The SARS-CoV-2 3CLpro is one of the primary targets for designing new and repurposing known drugs. **Methodology:** A virtual screening of molecules from the Natural Product Atlas was performed, followed by molecular dynamics simulations of the most potent inhibitor bound to two conformations of the protease and into two binding sites. **Conclusion:** Eight molecules with appropriate ADMET properties are suggested as potential inhibitors. The greatest benefit of this study is the demonstration that these ligands can bind in the catalytic site but also to the groove between domains II and III, where they interact with a series of residues which have an important role in the dimerization and the maturation process of the enzyme.

## Graphical abstract

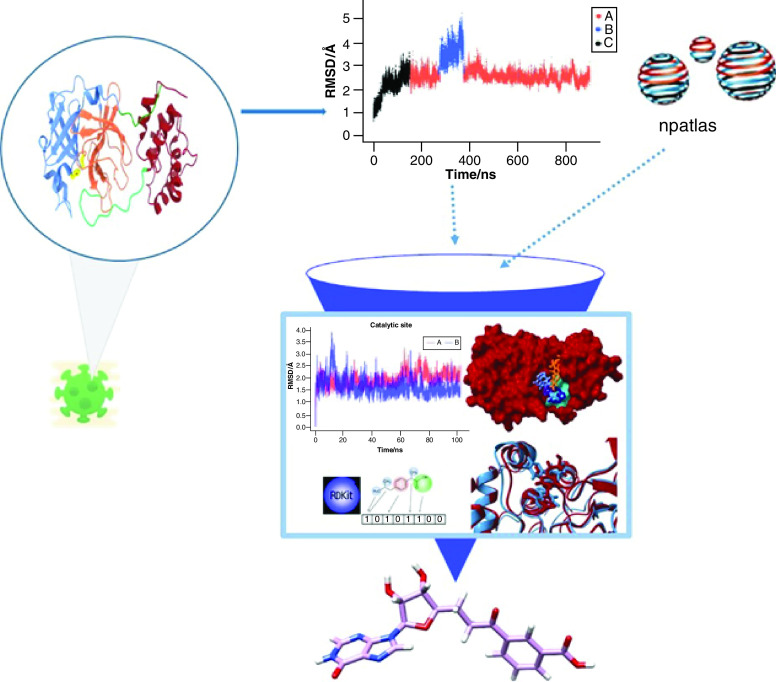

Since the first infections with SARS-CoV-2, a member of the coronavirus family, in December 2019 in Wuhan, China, more than 57.8 million people worldwide have been infected as of 22 November 2020 [1]. Unfortunately, at the time of writing, there were no available drugs against coronaviruses (CoV) according to WHO, the US FDA and the NIH [2–4].

The 33.8 kDa main protease named 3C-like proteinase (3CLpro), has been identified as a potential anti-CoV target, due to its crucial role in mediating viral replication and transcription [5–11]. The 3CLpros isolated from SARS-CoV and SARS-CoV-2 viruses have a 96% similarity in their primary sequences of 306 residues. The protein's 3D structure is divided into three domains (Figure 1). A structural motif characterizing domains I (residues 8–101) and II (residues 102–184) is a six stranded anti-parallel β-barrel, with four short α-helices. The binding site is located in the cleft between the two domains, with conserved residues His 41 (α2-helix, domain I) and Cys 145 (β11-sheet, domain II). A flexible long loop (residues 185–200) is connecting a globular cluster of five α-helices (domain III, residues 201–306) with the domain II [12]. Because in normal conditions 3CLpro dimerizes, Fan *et al.* [13] investigated the monomer–dimer equilibrium in solution and found that only the dimer form shows the catalytic activity. Chen *et al.* [14] performed enzyme activity experiments and molecular dynamics simulations of the hybrid SARS 3CLpro protein and concluded that the monomers on their own are unable to establish normal enzymatic activity, and only one protomer in the asymmetric homodimer has the correct conformation to perform catalysis. Shi and Song [15] conducted a series of experiments on site-directed mutants of SARS 3CLpro, investigating the monomer-dimer equilibrium and enzymatic activity. They identified four regions associated with the enzyme dimerization – residues 1–5 from the *N*-terminus forming the *N*-finger, residue Asn 214, the region around residues Glu 288–Asp 289–Glu 290 in a close contact with the *N*-finger, and the *C*-terminus’ last helix region around residues Arg 298–Gln 299. Molecular modeling and mutagenesis studies of the main protease of transmissible gastroenteritis coronavirus identified interactions involving the *N*-terminus and the α-helix domain responsible for the stabilization of the loop in the orientation required for the trans-cleavage activity [16]. Lim *et al.* demonstrated that Arg298Ala mutation in the SARS main protease completely stops the dimerization, resulting in an inactive monomeric form of the enzyme. On the other hand, a triple Ser284Ala, Thr285Ala and Ile286Ala mutant has a slightly enhanced dimerization capability and shows a 3.6-fold increase in the activity, compared with the wild-type protease [17]. Two mutations (Thr285Ala and Ile286Leu) observed in SARS-CoV-2 lead to a closer packing of the dimer with a slight increase in the catalytic efficiency, but without influencing the dimer dissociation [18].

**Figure 1.**
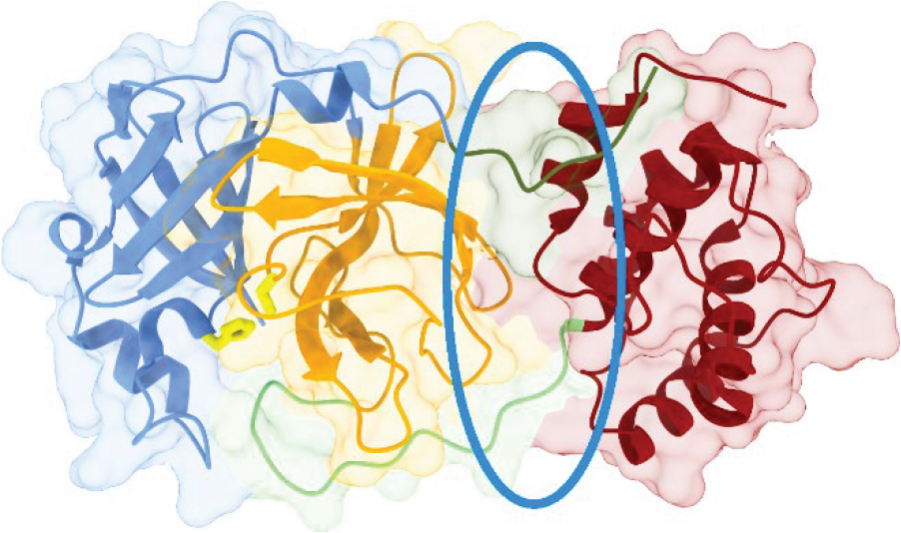
Structure of SARS-CoV-2 3CLpro (PDB ID: 6LU7). The *N*-finger (residues 1–7) is depicted in dark green, domain I (residues 8–101) in blue, domain II (residues 102–184) in orange, the loop region (residues 185–200) in light green, domain III (residues 201–306) in red, conserved His 41 and Cys 145 residues are in yellow, and the groove between domains II and III is encircled by blue ellipse.

Since the outbreak of SARS-CoV in March 2003 [19], scientists have been trying to repurpose existing drugs and develop a new generation of antiviral drugs. Both scientific and pharmaceutical communities got a fresh dose of motivation due to the global crisis caused by the SARS-CoV-2 pandemic. According to the US National Library of Medicine on 26 November 2020, there are currently 158 active and more than 1100 recruiting clinical trials worldwide connected to SARS-CoV-2 [20]. Most of those trials are testing performance of drugs that have already been approved or are in experimental phases for other diseases.

From a theoretical side, Li *et al.* conducted a virtual screening of 8000 drugs from available libraries, with SARS-CoV 3CLpro as the receptor. They identified prulifloxacin, bictegravir, nelfinavir and tegobuvir as safe and potential 3CLpro inhibitors. While prulifloxacin binds to the active site, other compounds have higher binding potential for the joint groove site [21]. Preliminary results of Sekhar's virtual screening of 3639 approved drugs and the SARS-CoV-2 3CLpro suggest that saquinavir and beclabuvir are also potential candidates for COVID-19 therapy [22].

Insilico Medicine [23] used its generative chemistry pipeline to design novel drug-like inhibitors. Their approach included a crystal-derived pocket-based generator, a homology modeling-based generation and a ligand-based generation. By 15 April 2020, 97 novel potential inhibitors of SARS-CoV-2 main protease had been generated. ul Qamar *et al.* adopted a different approach [24]. After constructing a 3D homology model, they screened it against a medicinal plant library containing phytochemicals and traditional Chinese medicinal compounds. Among 32,297 phytocompounds, 5,7,3′,4′-tetrahydroxy-2′-(3,3-dimethylallyl) isoflavone, an isoflavone extracted from *Psorothamnus arborescens*, had the highest binding activity due to a formation of strong hydrogen bonds with the conserved His 41 and Cys 145 residues, and numerous interactions with other residues from the catalytic site. Plenty of studies tested various plant and algae extracts for antiviral activity, including against SARS-CoV [25–30], demonstrating the antiviral potential of natural-based products. Those findings, together with a successful application of molecular docking for virtual screening of natural product databases [31–33], motivated us to virtually screen microbial natural products from the Natural Products Atlas [34] as potential SARS-CoV-2 3CLpro inhibitors.

## Methods

### Molecular dynamics simulation of the unbound protein

The SARS-CoV-2 3CLpro structure was obtained from RCSB (code 6LU7) [9]. Ligand and water molecules were removed, and amino acid residues were visually inspected. The protein was treated using the AMBER ff14SB forcefield [35] and was solvated in a truncated octahedral box of TIP3P water molecules spanning a 12 Å thick buffer. A total of 18,605 water molecules were added. The protein was then neutralized by 4 Na^+^ ions and submitted to geometry optimization in the AMBER16 program, employing periodic boundary conditions in all directions [36]. This was done in two steps: the first step included 470 cycles of the steepest descent method, followed by 1030 steps of the conjugate gradient method (a total of 1500 cycles). In this step the complex was restrained (*k* = 10.0 kcal mol^-1^ Å^-2^) and only the water molecules were optimized. The second step included 1000 cycles of the steepest descent method, followed by 1500 cycles of the conjugate gradient method (a total of 2500 cycles), where both the water molecules and the protein were unrestrained. The minimized systems were slowly heated from 0 to 300 K during 30 ps under NVT conditions. This was followed by the unconstrained molecular dynamics (MD) simulations (900 ns) with a time step of 2 fs, with pressure (1 atm) and temperature (300 K) held constant. For the temperature control, the Langevin thermostat with a collision frequency of 1 ps^-1^ was used. Bonds with hydrogen atoms were constrained using the SHAKE algorithm [37], the long-range electrostatic interactions were computed using the Particle Mesh Ewald method [38], and the non-bonded interactions were truncated at 11.0 Å. After the simulation, the first 150 ns of the simulation were disregarded, ensuring only the stable protein conformations were used (a total of 375,001 trajectory snapshots were kept). A *k*-means cluster analysis based on all nonhydrogen backbone atoms was performed using CPPTRAJ [39], with maximal number of iterations set to 500, randomized initial set of points used and sieving set to 10. The number of clusters was found to be two, based on the Davies-Bouldin index (DBI) and the pseudo F-statistic (pSF) (Supplementary Tables 1 & 2) [40]. After clustering, frames closest to the centroids of each cluster were identified as the two different conformations and were used as receptors in docking studies. The clustering analysis was done for two simulation runs with different random seeds in order to confirm the results.

MD simulations were performed on the Isabella cluster of University Computing Center of University of Zagreb, Croatia, based on NVIDIA Tesla V100 GPU.

### Molecular docking

A database of microbial natural products was obtained from the Natural Products Atlas web page (version v.2019_12) [34,41] containing referenced data for structures, compound names, source organisms and other information for 25 523 molecules. SMILES notations of the molecules were converted to 3D structures, optimized and converted to pdb files using Chimera 1.14 [42]. AutoDockTools 4 script prepare_ligand4.py [43] was used to prepare the molecules for docking, saving them in the pdbqt file format. To filter out too large and too flexible molecules, two criteria were introduced: molecular mass threshold (700 Da) and the number of rotatable bonds (less than 15); 19,250 molecules satisfied these criteria. The molecular weight distribution and the number of rotatable bonds distribution for the Natural Products Atlas molecules are displayed in Supplementary Figure 3. Only neutral forms were considered. Docking experiments were performed on South Ural State University's supercomputer based on Intel Xeon X5680 processors @3.3 GHz. All structures were docked to the two conformations representing the two clusters obtained by the aforementioned cluster analysis. The receptor molecules were prepared using Chimera 1.14. Gasteiger charges were added to each atom, nonpolar hydrogens were merged, atom types were determined and structures of the prepared receptors were saved as pdbqt files. The two conformations obtained from the clustering were superimposed based on the active site and the center of the grid box was the Cys 145 CA atom, with Cartesian coordinates 13.3, 58.2 and 45.4, whereas the size of the box was 20 × 25 × 25 Å. Number of modes and exhaustiveness were both set to 100. Docking was performed using the AutoDock Vina software [44]. The weighted binding score (*K*_w_) for each molecule was calculated using the formula:

(Eq. 1) $$K_{W}=0.867\times K_{1}+0.133\times K_{2}$$

where *K*_1_ and *K*_2_ are scores for the receptor in the primary and the secondary conformation (as calculated by the clustering algorithm), weighted by the fraction of time receptor spends in that conformation during the simulation time. The graph-based signature approach, as implemented on the pkCSM web server [45], was used to predict ADMET properties of the top 200 hit molecules. For 8 molecules with the best binding scores and whose ADMET properties classified them as potential drug candidates, blind docking experiments were run to check for alternative binding sites outside the active pocket. In this case, the receptor's center of the mass was the origin of the grid box (coordinates 24.1, 48.8, 48.2), with box dimensions of 56 × 52 × 58 Å. Both the number of modes and the exhaustiveness were set to 100. For each ligand, all conformations within 4 kcal mol^-1^ relative to the conformation with the highest score were saved and the conformation with the lowest binding energy bound to the pocket of interest was kept (after visual inspection of the plausibility of the said conformation). The conformations of hit molecules with the best score outside the catalytic pocket were used to analyze the potential of the newly identified groove binding site (Figure 1).

### Fingerprinting and molecular similarity

The 2D molecular fingerprints (FP) were calculated for all eight hit molecules. RDKit (2020.03.1) [46] was used for FP and molecular similarity calculation. In the present study the Morgan circular (i.e., the extended connectivity) [47] FP were generated, with the radius of the fingerprint set to four. Then, the molecular similarity calculation based on the Tanimoto coefficient was performed [48]. The range of the Tanimoto coefficient, *T_c_*, varies from zero to one, where 0 represents minimum and 1 maximum similarity.

The generated FP for natural products were compared to the calculated FP for 8750 molecules from the DrugBank database (Release Version 5.1.5) [49]. Additionally, the FP similarities were evaluated for futalosine against 1,914,278 molecules from the ChEMBL database (Release Version ChEMBL 27) [50]. Both databases hold curated and experimentally validated information. In total, 5543 compounds from ChEMBL are targeting SARS-CoV-2, and for them FP similarities were calculated against all top eight hit molecules.

### MD simulation of the bound protein

Initial complex conformations for MD simulations were obtained by docking experiments. For the two conformations of the main protease (**A** and **B**) natural product futalosine was bound either in the active or in the groove site. Futalosine was parametrized using the AMBER 16 Antechamber module [51] and the GAFF [52] forcefield. For the protein, the AMBER ff14SB [35] was employed. Such complex was then neutralized by sodium ions and solvated in a truncated octahedral box with TIP3P [53] water molecules spanning a 12-Å-thick buffer. Simulation protocol was identical as for the unbound protein, with only difference being the total simulation time, which was in this case 100 ns.

Simulation analysis was performed using CPPTRAJ [39]. The Python written MMPSBA.py program [54] was used to calculate the binding free energies, Δ*G*_bind_, of the futalosine–3CLpro complexes using the established MM/GBSA protocol [55,56]. For that purpose, 2500 snapshots collected from the last 50 ns of the corresponding MD trajectories sampled at regular time steps were used. The calculated MM/GBSA binding free energies were decomposed into specific residue contribution on a per-residue basis according to the established procedure [57,58]. Entropic contributions (-*T*Δ*S*) were neglected.

## Results & discussion

### MD simulations – SARS-CoV-2 3CLpro

**Figure 2.**
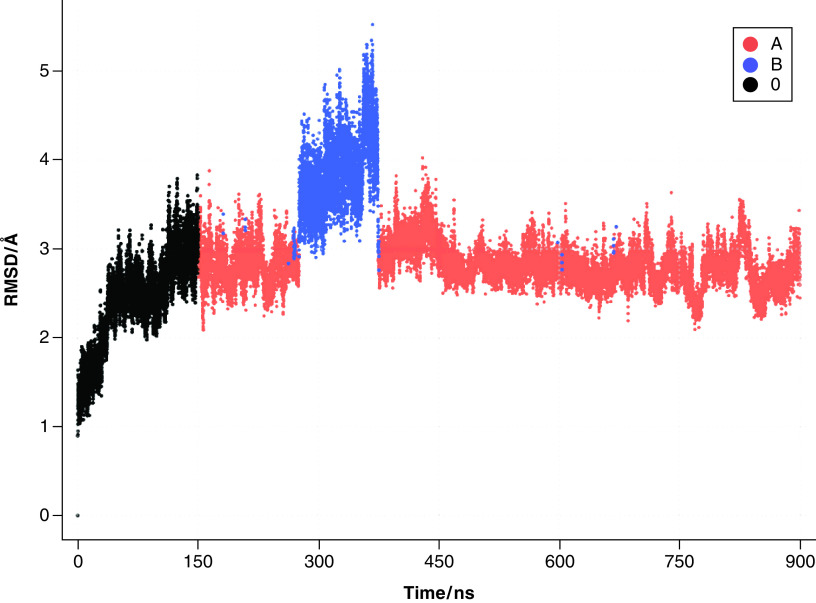
Evolution of the free protein backbone root mean square deviation with clusters shown.

Evolution of the free protein backbone root mean square deviation (RMSD) is shown in Figure 2. It can be seen that two main protein conformations are present (the primary conformation A and the secondary conformation B, which are present 86.7 and 13.3% of the simulation time, respectively), with the first 150 ns of the simulation (equilibration phase, black dots) disregarded. RMSD between the two cluster representatives is 6.77 Å. Details about alternative clusters, as well as the repeated MD simulation are presented in Supplementary Tables 1 & 2 in the Supplementary information.

**Figure 3.**
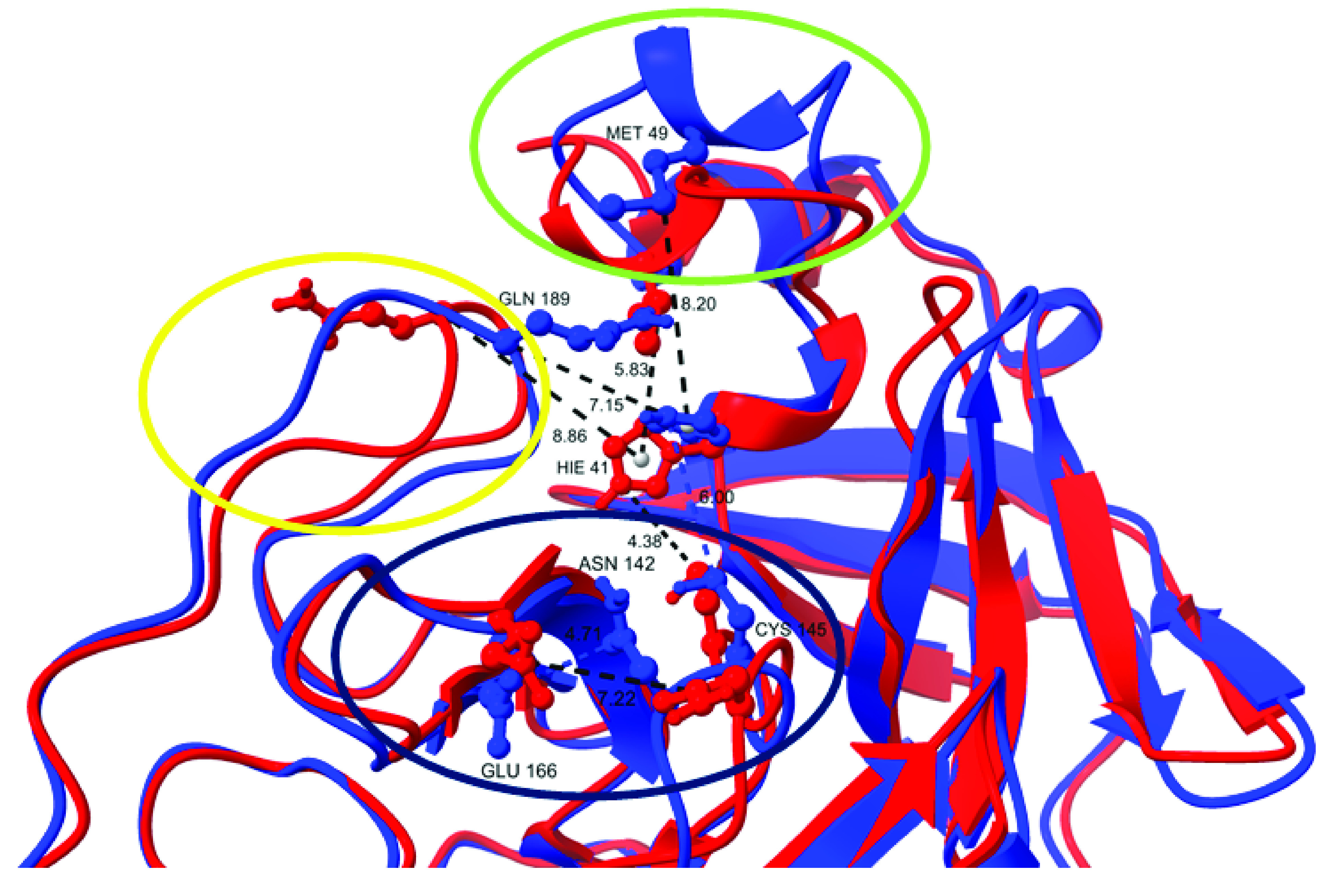
Overlaid structures of the representative primary (red) and secondary (blue) conformations of the SARS-CoV-2 3CLpro extracted from the molecular dynamics simulation, with distances (in Å) between the selected residues and encircled areas with major structural rearrangements.

Structural differences in the binding site geometries of the two conformations are significant (Figure 3). In the catalytic dyad, composed of the His 41 and the Cys 145 residues, in the conformation **A**, the distance between the Cys 145 sulphur atom and the His 41 NE2 type atom is less than 4.38 Å, while the analogous distance in the conformation **B** is 6.00 Å. The orientations of the His 41 imidazole ring in the **A** and **B** conformations are almost perpendicular to each other, influencing the depth of the binding pocket. This might be a consequence of structural differences in the three areas in the vicinity of the active site. Additionally, the unstructured sequence (from Ile 43 to Glu 47) continuing to the short helix (Asp 48-Met 49-Leu 50) (Figure 3, green ellipse) is highly flexible. Met 49 SD atom type is 5.83 Å away from the centroid of the His 41 imidazole ring in the **A** conformation. This distance increases to 8.20 Å in the **B** conformation, due to the conformational changes and the movement of the helix away from the catalytic dyad. The sequence from Phe 185 to Gln 192, which makes up the flexible and unstructured loop, experiences major structural rearrangements in the two conformations: the Gln 189 side chain is facing toward His 41 in the **B** form (the distance between Gln 189 CA atom and His 41 centroid is 7.15 Å), lying between His 41 and Met 49, while in the **A** form it is exposed to the solvent (Figure 3, yellow). The Ser 139-Phe 140-Leu 141 loop, whose transformation to the 3_10_-helix inactivates the catalysis [59], also experiences structural changes that modify the active site geometry (Figure 3, dark blue). Another difference is the Asn 142 amino group, which faces the β-sheet in the **B** conformation. This interaction seems to have a stabilizing effect, with the distance between Asn 142 and Glu 166 CG atom types being only 4.71 Å. However, in the **A** conformation, this interaction is missing due to the solvent exposed orientation of the Asn 142 side chain.

All these structural changes lead to a modification of the active site geometry, influencing its depth and width (Supplementary Figure 1). Because the clustering was based on the RMSD of the backbone atoms only, it did not capture the dynamics of the side chains. To get more details of the geometric changes of the catalytic site, we analyzed the four aforementioned distances during the simulation time (Supplementary Figure 2), with the first 150 ns of the simulation disregarded. The relationship between the catalytic dyad residues is very important for 3CLpro's activity. The fact that the distance between the Cys 145 SG atom and the centroid of the His 41 imidazole ring is longer for the **A** conformation (5.38 Å) than for the **B** conformation (4.55 Å) means that the catalytic pocket is deeper in the **A** conformation. Also, fluctuations are more evident in the **A** conformation (standard deviation is 0.95 Å), whereas in the **B** conformation it is ‘locked’ (standard deviation is 0.38 Å). Additional comparison of the crystallographic structure and the two conformation can be found in Supplementary Information (Supplementary Figure 4 & Supplementary Table 4).

The 3CLpro activity regulation is still not fully understood. Comparing the active and the inactive protomers of SARS-CoV 3CLpro, Li *et al.* pinpointed to the Ser 139-Phe 140-Leu 141 loop, whose transformation to a short 3_10_-helix disrupts the catalytic machinery in the inactive monomer structure [59]. Our analysis showed that the mean distances between the Asn 142 CG atom types (close to both the Cys 145 residue and the Ser 139-Phe 140-Leu 141 fragment) and Glu 166, as well as the distance between the His 41 ring centroid and the Gln 189 CA atom are shorter in the **B** conformation, making the **A** conformation's catalytic pocket wider on average. The greatest difference is in the mean distances between the His 41 centroid and the Met 49 SD atom type, where the position and the orientation of the short helix influences the His 41 imidazole ring orientation.

### Docking experiments

The microbial natural products from the Natural Products Atlas that satisfied selection filters were docked to two SARS-CoV-2 3CLpro conformations, obtained by a MD simulation (as described in the previous section). The weighted docking score was calculated according to Equation 1. The average weighted docking score was 6.1 ± 0.9 kcal mol^-1^.

To further filter the potential hit molecules, we implemented criteria based on ADMET properties: not a cytochrome P450 inhibitor (predicted for CYP1A2, CYP2C9, CYP2D6, and CYP3A4 enzymes), not a P-glycoprotein I/II inhibitor, nontoxic (not an inhibitor of hERG I/II), noncancerogenic (AMES negative) and nonhepatotoxic. Besides the constrains imposed on molecules from the Natural Products Atlas to qualify for docking (molecular weight and number of rotatable bonds), additional criteria for oral bioavailability were also implemented: lipophilicity (logP) <5; hydrogen bond acceptor sites ≤10; and 3) hydrogen donor sites ≤5. After employing all these criteria, top eight molecules with best docking scores satisfying all criteria were collected in Table 1 (the complete list with all the tested molecules can be found in Supplementary Table 3).

**Table 1.** Hit molecules from the Natural Products Atlas, as potential SARS-CoV-2 3CLpro inhibitors.

ATLAS ID (alternative name)	2D structure	*M*_r_	*N*_rot_	*N*_acc_	*N*_don_	log*P*	*K*_1_	*K*_2_	*K*_w_
NPA013652 (19-hydroxypenitrem A)		650.2	1	7	5	4.78	-9.4	-7.1	-9.1
NPA001702 (2′,3′-Epoxymyrothecine A)		518.6	0	10	3	0.66	-8.8	-7.3	-8.6
NPA002809 (Futalosine)		414.4	6	9	4	0.10	-8.6	-7.9	-8.5
NPA005589 (Pseudonocardone C)		510.5	4	10	5	0.91	-8.7	-6.8	-8.4
NPA022742 (MDN-0185)		507.5	0	10	4	0.96	-8.5	-8.1	-8.4
NPA013618 (Pityriarubin A)		526.5	3	4	5	4.69	-8.5	-7.9	-8.4
NPA015941 (Izumiphenazine B)		468.4	2	9	4	3.45	-8.2	-8.4	-8.2
NPA010921 (Verrucarin Y)		514.5	1	9	1	2.18	-8.2	-8.2	-8.2

*M*_r_: Molecular weight; *N*_acc_: Number of hydrogen bond acceptor sites; *N*_don_: Number of hydrogen bond donor sites; log*P*: Lipophilicity; *K*: Docking score (in kcal mol^-1^).

**Figure 4.**
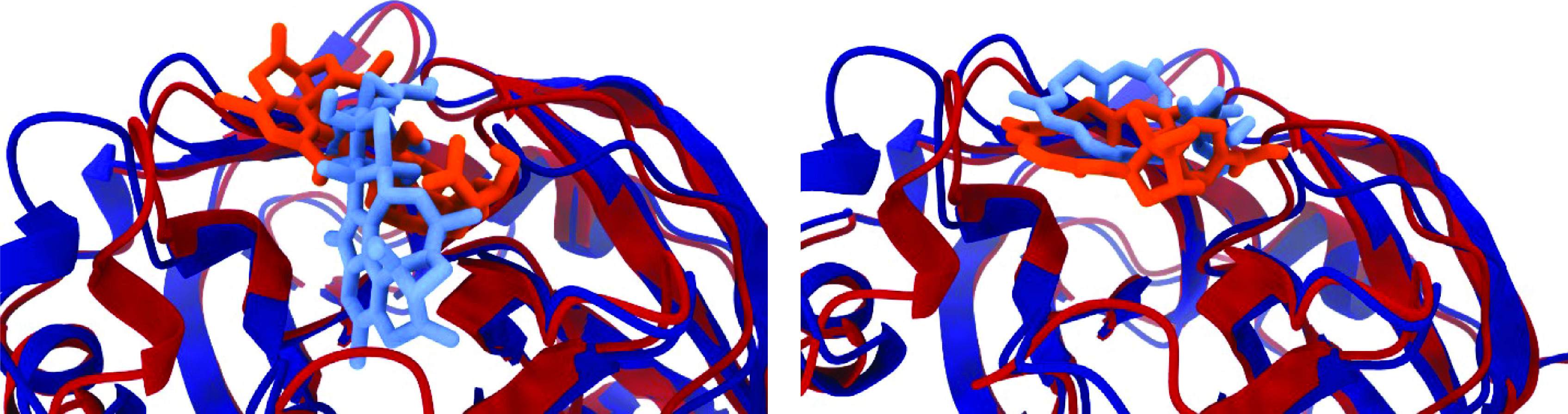
An overlay of docked 19-hydroxypenitrem A (left) and verrucarin Y (right) complexes to the A (red) and B (blue) SARS-CoV-2 3CLpro conformations.

**Figure 5.**
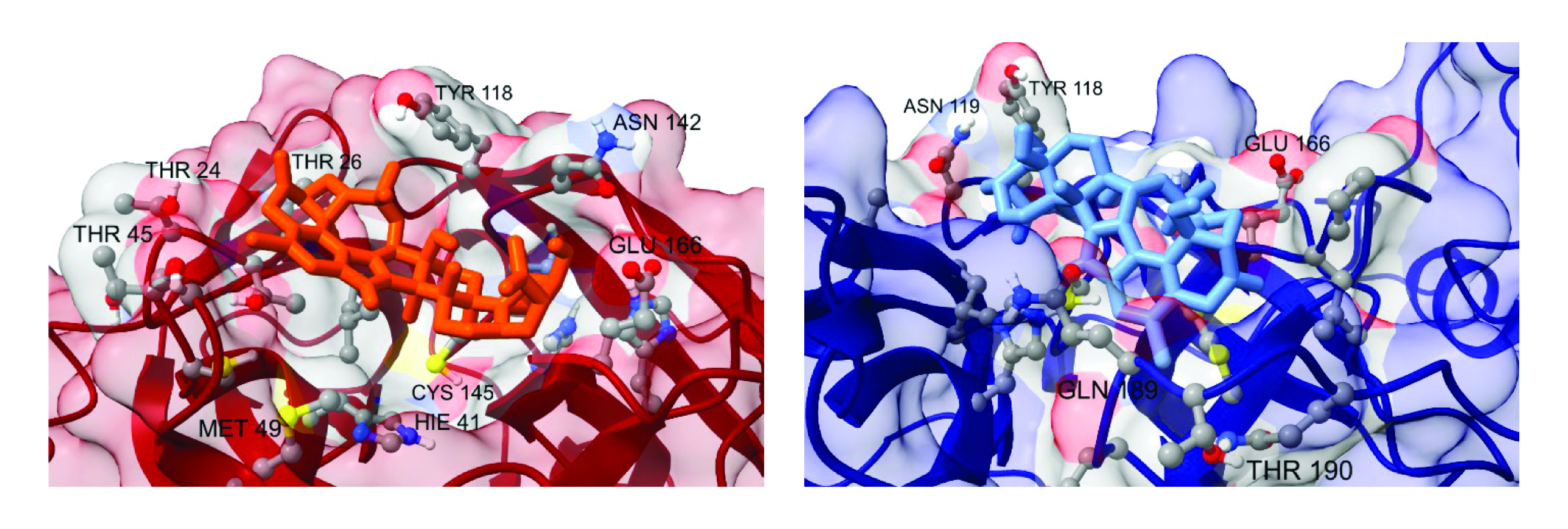
Insight into the catalytic site neighborhood for two conformations of the 19-hydroxypenitrem A – SARS-CoV-2 3CLpro complex: A (left) & B (right).

It is interesting to note very different binding free energies of 19-hydroxypenitrem A to the **A** and **B** conformations of the SARS-CoV-2 3CLpro. A comparison of the docked complexes already provides an important piece of information about interactions between the ligand and the receptor, revealing the nature of the observed phenomenon (Figure 4, left). 19-hydroxypenitrem A docks to the **A** and **B** conformations following different patterns. In the **A** conformation, it is bound at the surface of the active pocket, while in the **B** conformation it penetrates deeper into the pocket (Figure 5). On the other hand, verrucarin Y has identical binding free energies for both the **A** and **B** conformations (Table 1). This can be explained by a similar binding to the catalytic pocket, with minor translations and rotations of the ligand at the surface of the pocket (Figure 4, right).

**Figure 6.**
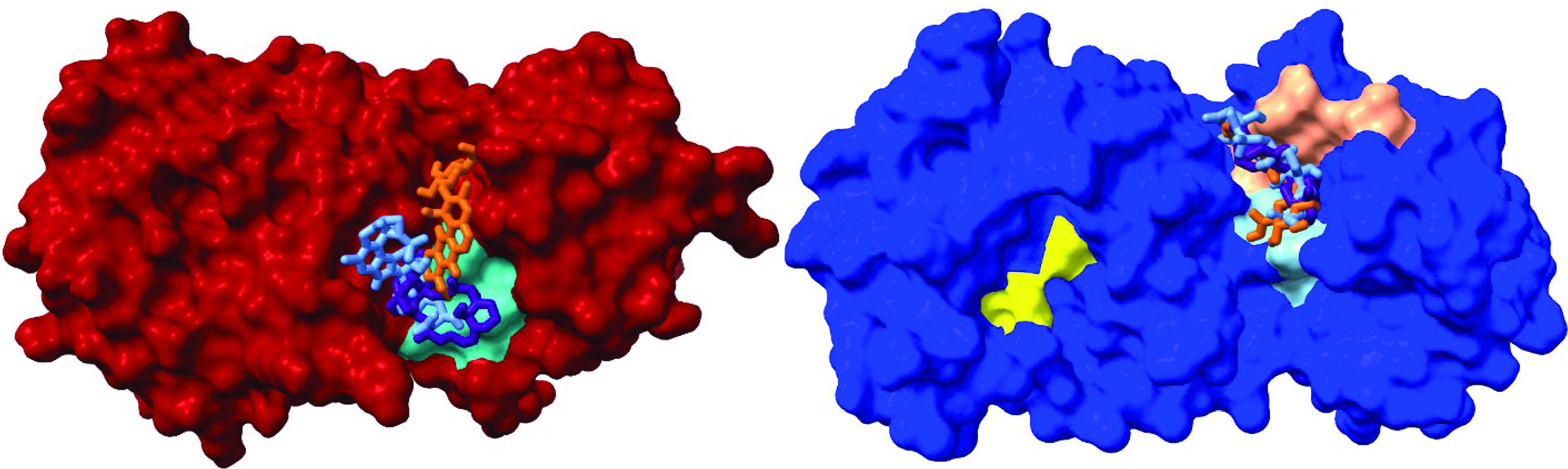
A new SARS-CoV-2 3CLpro groove binding site in conformations A (left, red) and B (right, blue) in complexes with hit molecules 19-hydroxypenitrem A (light blue), 2′,3′-epoxymyrothecine A (indigo) and pseudonocardone C (orange). His 41 and Cys 141 residues are depicted in yellow, the *C*-terminus’ last helix region in cyan, the *N*-finger in tan and residues 288–290 in light blue.

The blind docking results for the eight most promising SARS-CoV-2 main protease inhibitors identified a potential allosteric binding site. When the hit molecules are docked to the **A** conformation, in all cases the ligands with the lowest binding free energies are located in the active site. However, in the **B** conformation, which is present for 13% of the MD simulation, a new binding site emerges in the groove between domains II and III (Figure 6, left). In this case, binding free energies are slightly lower, ranging from -7.8 kcal mol^-1^ for futalosine to -9.0 kcal mol^-1^ for 19-hydroxypenitrem A (Table 2). A closer analysis reveals that ligands bound in this groove site are interacting with the *C*-terminus’ last helix region, which is known to be associated with the 3CLpro dimerization [15]. This finding increases a potential of hit molecules as main protease inhibitors by dual mechanism. First, by binding to the catalytic site, they prevent substrate binding. Second, a formation of an inhibitor–enzyme complex, where an inhibitor is bound in the groove site, could prevent the maturation of the enzyme (i.e., its dimerization). The groove binding site is loosely defined. It includes several regions between domains II and III, and binding of a potential inhibitor in some of these regions gives similar binding free energies. In this study, we concentrated on the region in the groove binding site which corresponds to the lowest binding free energy. For the **B** conformation (the conformation with a lower population), the groove geometry is considerably different than for the **A** conformation (Supplementary Figure 5). If domains I and II of the SARS-CoV-2 3CLpro are overlaid, one can easily see the movement of the domain III, connected to the domain II by a flexible and unstructured loop. In the **B** conformation, a new binding site is identified (Figure 6), with a higher binding constant for the tested molecules, compared with the catalytic site. Here, the ligands are bound next to the *N*-finger (residues 1–5), which is believed to have an important role in the dimerization process [15]. The key interaction in the dimerization process is believed to be the interaction between the Arg 4 residue of the first protomer and the Glu 290 residue of the second protomer. This interaction is present on both sides of the dimer [18].

**Table 2.** The predicted binding free energies (K) for the eight hit molecules to the SARS-CoV-2 3CLpro in the groove binding site.

ATLAS ID (alternative name)	*K*_1_	*K*_2_	*K*_w_
NPA013652 (19-hydroxypenitrem A)	-9.0	-8.6	-8.9
NPA001702 (2′,3′-epoxymyrothecine A)	-8.0	-8.6	-8.1
NPA002809 (futalosine)	-7.8	-8.5	-7.9
NPA005589 (pseudonocardone C)	-7.9	-8.6	-8.0
NPA022742 (MDN-0185)	-8.4	-9.7	-8.6
NPA013618 (pityriarubin A)	-8.3	-9.0	-8.4
NPA015941 (izumiphenazine B)	-8.1	-8.9	-8.2
NPA010921 (verrucarin Y)	-8.0	-8.7	-8.1

### Fingerprint and molecular similarity analyses

It is a difficult task to choose top hits molecules from a virtual screening experiment without having experimental data or additional computational calculations. In this concern, several computational techniques are available to filter out lead molecules, among which the ADMET prediction is the most widely used. In a virtual screening approach, when a large number of molecules is tested, the ADMET filter is not enough. This problem can be overcome by employing the similarity-based virtual screening method; however, only if known inhibitors are available against the target of the interest [60,61]. The main concept behind the similarity-based virtual screening is the structure–activity relationship (SAR) – molecules with similar structures have a similar biological activity. Because currently there are no approved drugs against 3CLpro, we applied a molecular similarity search in the lead discovery step against the DrugBank molecules.

All the top eight hits were subjected to a 2D molecular fingerprint (FP) analysis using the Morgan circular, that is, the extended connectivity FP (ECFP), to check for their similarity with DrugBank molecules. Previously, several reports highlighted the importance of the FP method selection for virtual screening, and analyzed the difference in the results obtained by different FP approaches [62,63]. Riniker *et al.* reported that ECFP offers the highest precision on average, according to database search by compound similarity based on FP [64]. Hence, in our analysis, ECFP was used for the identification of the top lead molecule based on its resemblance to the DrugBank molecules. The release of DrugBank used in the present study (version 5.1.5) contains almost 9000 drug entries including approved small molecule drugs, approved biologics, nutraceuticals and experimental drugs [65]. Futalosine, a secondary metabolite isolated from the *Streptomyces* genus of Actinobacteria, was found to have the highest Tanimoto molecular similarity index (*T*_c_ = 0.489), suggesting its structural similarity with already approved and experimental drugs. Due to its drug-likeness, futalosine was then selected for further molecular dynamics studies. It shows the highest similarity index with the DrugBank molecules DB05973 (inosine 5′-sulfate) and DB04566 (inosinic acid). Inosinic acid is a purine nucleotide with hypoxanthine as the base. Futalosine can be considered as a derivative of the inosinic acid, where the 5′-monophosphate group on the tetrahydrofuran-3,4-diol moiety has been substituted by the 3-butanoylbenzoic acid. Recently, one of inosine derivatives, inosine pranobex, was approved for clinical trials against the SARS-CoV-2 virus (identifiers NCT04383717 and NCT04360122) [20]. Molecular similarities for other hit molecules are presented in Supplementary Table 5.

The ChEMBL database holds manually curated bioactive molecules with drug-like properties and it also brings together chemical, bioactivity and genomic data to aid the effective translation of new drugs. To check whether any similar molecules/scaffolds are under investigation against any type of diseases, similarity search for futalosine was also performed against molecules from the ChEMBL database. Molecules under IDs CHEMBL3246765 (*T*_c_ = 0.492) and CHEMBL1198613 (*T*_c_ = 0.491) showed the highest similarity to futalosine among the 1 914 278 collected ChEMBL molecules when circular FP are compared. They all share the same hypoxanthine motif attached to the tetrahydrofuran-3,4-diol moiety (CHEMBL3246765). CHEMBL1198613 additionally has a *N*-(2-hydroxybenzoyl) sulfamate unit. The CHEMBL3246765 [66] molecule is currently under investigation for inhibition of human erythrocyte purine nucleoside phosphorylase, and chemically modified CHEMBL1198613 is investigated against *Mycobacterium tuberculosis* [67]. The eight hit molecules were tested against the potential SARS-CoV-2 inhibitors from the ChEMBL database, and again futalosine obtained the highest Tanimoto coefficient. More details for FP of the ChEMBL database are provided in the supplementary information (Supplementary Figures 6 & 7).

### MD simulations – the SARS-CoV-2 3CLpro: futalosine complex

**Figure 7.**
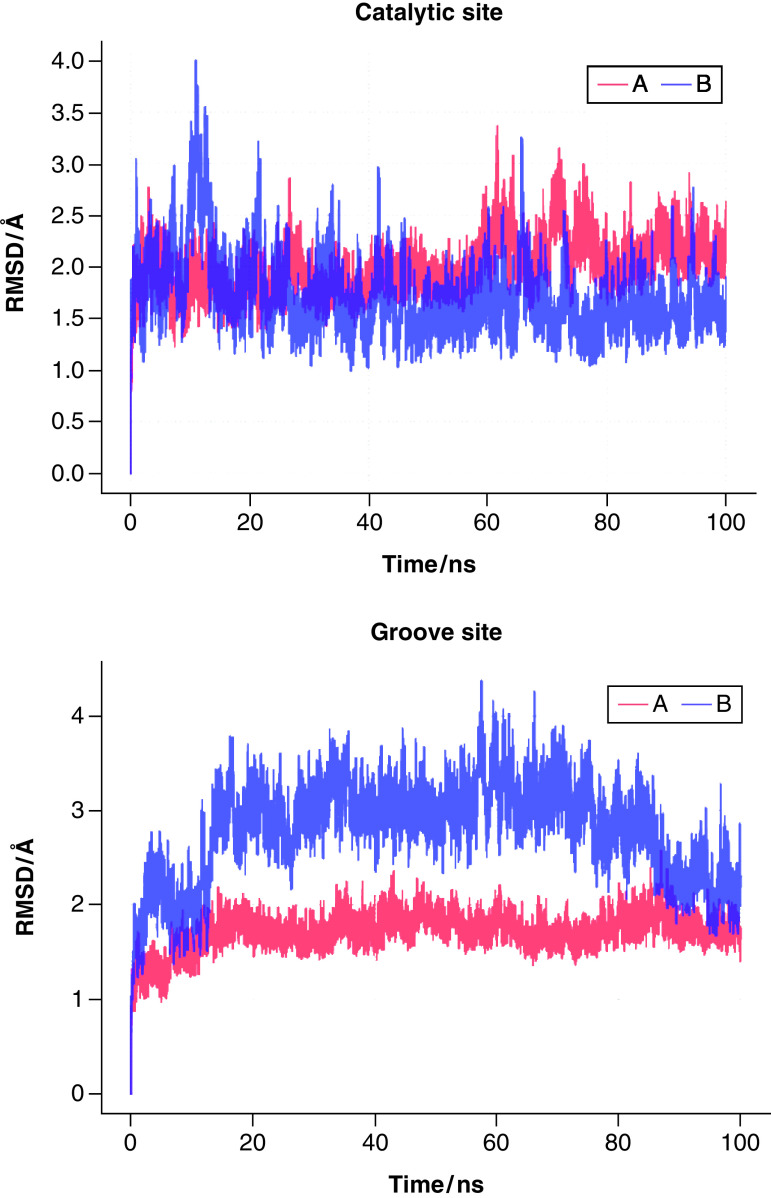
Evolution of the root mean square deviation of SARS-CoV-2 3CLpro–futalosine complex based on the whole backbone. Futalosine bound in the catalytic site (left) and in the groove site (right) for main protease conformations **A** (red) and **B** (blue).

To check the stability of the docked complexes, 100 ns MD simulations were conducted for the SARS-CoV-2 3CLpro–futalosine complex. We opted for this particular ligand due to its drug-likeness, as described in the previous section. From the RMSD evolution of the main protease complex with futalosine either in the catalytic or in the groove binding site, it can be seen that both complexes are stable and are not dissociating on the time scale of our simulations. When the ligand is bound to the catalytic site of the **A** conformation (Figure 7, left), it experiences a reorientation within the binding pocket at around the 60 ns mark of the simulation. This change is also reflected in an increase of the mean RMSD value from 1.9 Å, for the first 60 ns, to 2.0 Å for the last 40 ns. The benzoic acid moiety, which is perpendicular to the hypoxanthine fragment in the docked structure, reshuffles. It is positioned just above the His 41 residue, establishing aromatic interactions, while being partially shielded from the solvent by Ser 46. At the same time, the aromatic heterocycle lies between the two β-sheets. The reshuffling process influences the dynamics of the catalytic dyad residues (Supplementary Figure 8). At the beginning of the simulation, the Cys 145:SG – His 41:centroid distance is 4.58 Å, and it more than doubles within the first 10 ns of the simulation. Just after the 20 ns mark, the distance starts to oscillate around 6 Å. With futalosine bound to the catalytic site, the average Cys 145:SG – His 41:centroid distance is increased from 5.38 to 6.38 Å for the free protease. Futalosine molecule is bound to the **B** conformer in the opposite way (see Supplementary Figure 9). Here the hypoxanthine unit is locked between His 41 and Met 165, and the benzoic acid moiety interacts with Tyr 118, Asn 119, and Thr 26 residues. Throughout the simulations, the enzyme retained its starting conformation in both complexes.

**Figure 8.**
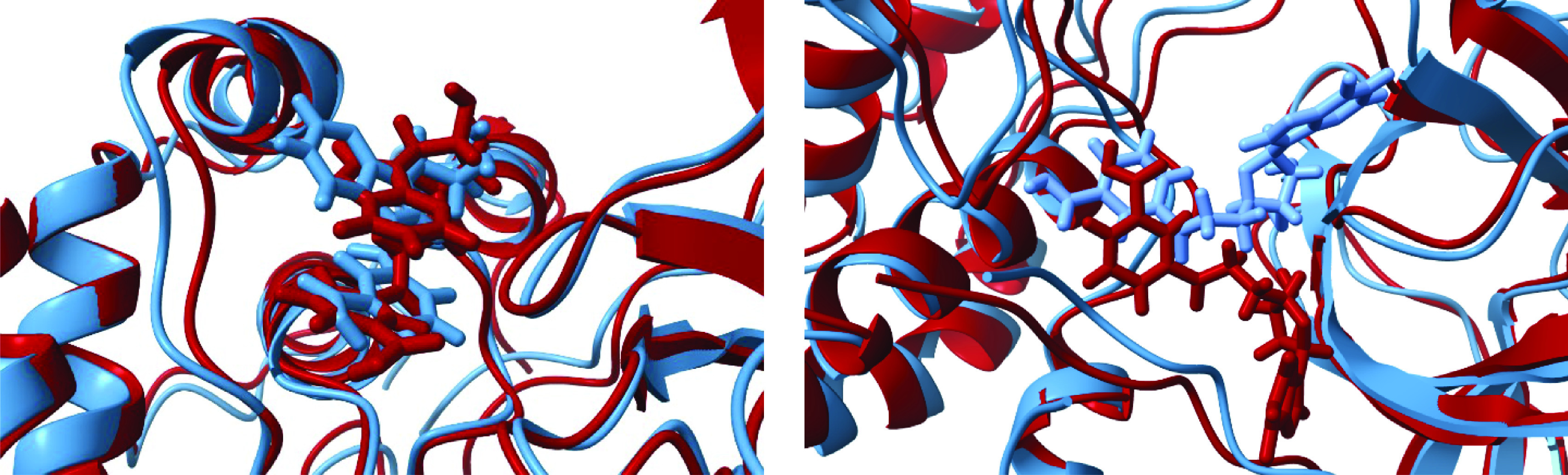
Comparison of structures from docking experiments (red) and the last structure from 100 ns molecular dynamics simulations runs (blue) for SARS-CoV-2 3CLpro conformations A (left) and B (right) with futalosine bound in the groove site.

Both conformations of the main protease–ligand complex where the ligand is bound in the groove site are stable on a 100 ns time scale (Figure 7, right). Comparing the initial (docked) and final structures from the MD simulations for the groove binding site (Figure 8), it can be seen that in the case **A**, the ligand retains its conformation with the intramolecular aromatic interactions. The hypoxanthine fragment is exposed to the solvent, and the benzoic acid moiety is hydrogen bonded to Glu 240. The movement of the tetrahydrofuran-3,4-diol fragment is constrained by two hydrogen bridges, with Gln 110 and Glu 240 residues. For the **B** conformation, the benzoic acid part of the ligand is anchored inside the groove. In the final structure, its carboxylic group forms three hydrogen bonds, with Phe 3, Trp 207, and Leu 282. Higher RMSD values for the **B** conformation can be explained by a higher ligand flexibility within the groove and the absence of the intramolecular aromatic interactions. It should be noted that Glu 290 could act as a hydrogen acceptor for hydroxyl hydrogens from the tetrahydrofuran-3,4-diol unit. The interaction pattern between SARS-CoV-2-3CLpro and futalosine in the groove binding site indicates a great potential of the groove binding site as a potential inhibition target. The ligand interacts and forms hydrogen bonds both with residues from the *N*-finger region and the region around residues Glu 288-Asp 289-Glu 290. Although the dimerization process is not fully understood, targeting the groove binding pocket between domains II and III might disrupt the necessary interactions between the two monomers, preventing dimerization and activation of the protease. It is believed that the salt bridge interactions between Glu 290 of one protomer and Arg 4 of the other protomer play a significant role in the dimerization process [18]. Second, the interactions between the *N*-fingers of each of the two protomers and the conserved Glu 166 residue of the other protomer promote the dimerization and help in shaping the pocket for substrate binding [16,18]. This alternative approach to carefully design molecules that would interact with these residues, which are critical for dimerization and the initiation of the catalytic activity, might provide a new way to fight SARS-CoV-2. An additional benefit would be if a second ligand molecule could efficiently bind to the catalytic site at the same time, decreasing the enzyme activity through two different mechanisms.

A logical question that follows is if (and how) the geometry of the catalytic site is affected by binding of the ligand to the groove site. Again, we analyzed the evolution of four selected geometrical parameters throughout the molecular dynamics simulations (Supplementary Figure 8). For the primary **A** conformation, the effect is small, but not negligible. Compared with the free SARS-CoV-2 3CLpro, the Cys 145:SG – His 41:centroid and Met 49:SG – His 41:centroid distances are 0.08 Å and 0.29 Å shorter, respectively. The other two average distances are 0.25 Å longer. This shows that futalosine has a potential to inhibit the main protease by the proposed dual mechanism. It binds well both to the catalytic and to the groove site. If it is bound to the groove site, it does not significantly influence the catalytic pocket structure, allowing a second molecule to occupy the catalytic site.

Ligand binding affinity was estimated by the MM/GBSA method, where the solvation free energies are calculated by solving the Generalized Born equation. The final binding free energies are a sum of van der Waals, electrostatic and polar and nonpolar contributions. Because we are comparing energies of the same enzyme and ligand, entropic contributions (-TΔS) was neglected. The final estimated binding free energies (without the unfavorable entropy contribution) of futalosine binding to the catalytic pocket of the **A** and **B** conformations are -33.7 ± 3.5 kcal mol^-1^ and -29.0 ± 3.8 kcal mol^-1^, respectively. Corresponding binding free energies when futalosine is bound to the groove site are -24.4 ± 3.3 kcal mol^-1^ and -30.1 ± 4.7 kcal mol^-1^.

A more detailed insight into the key residues with substantial contributions to the enzyme–ligand binding was enabled by the MM/GBSA binding free energy decomposition. A substantial contribution was defined as a contribution of a single residue to the binding free energy which is equal or lower than -1.5 kcal mol^-1^. Two residues (Asn 119 and Met 165) were found to have a substantial contribution in both **A** and **B** conformations, when the ligand was bound in the active site. Asn 119 was forming hydrogen bonds with the hypoxanthine ring or with the benzoic acid moiety, and Met 165 was establishing numerous van der Waals contacts with the ligand. Additionally, in the **A** conformation, Leu 27 and Tyr 118 also have significant contributions to the binding free energy. These residues are located on the opposite sides of the heterocyclic ring, making numerous van der Waals contacts and alkyl–aromatic interactions. These contacts are not present in the **B** conformation due to the different ligand orientation. However, in this case, two other residues become very important: Glu 166 forms hydrogen bonds with hydroxyl groups of the tetrahydrofuran-3,4-diol moiety, while Gln 189 is a hydrogen donor to the hypoxanthine carbonyl group. In the groove binding site, His 246 and Pro 108 have a dominant role in the **A** conformation, and Phe 3 and Glu 290 in the **B** conformation.

## Conclusion

In this article, a virtual screening of molecules from the Natural Products Atlas was performed, with SARS-CoV-2 3CLpro as the receptor molecule. A molecular dynamics simulation of the solvated ligand-free protease was done, after which a *k*-means cluster analysis was performed. Two enzyme conformations (with shifted domains III and significant differences in the catalytic site geometries) were obtained. After calculating and analyzing ADMET properties predictions and docking scores, eight potential hits were identified. Additionally, a blind docking procedure identified additional binding sites in the groove between domains II and III, where potential inhibitors interact with either the last helix at the *C*-terminus side (in the dominant, **A** conformation) or with the *N*-finger (in the **B** conformation). One of the found compounds, futalosine, demonstrated a good drug-likeness. The molecular dynamics simulations were performed for futalosine complexed with both SARS-CoV-2 3CLpro conformations. We found that, besides a site discovered experimentally, one more potential binding site exists in the groove between domains II and III, which could be exploited to prevent enzyme dimerization, leaving it in the immature and inactive state. We demonstrated that complexes of futalosine with both sites of SARS-CoV-2 3CLpro in both conformations are stable.

## Future perspective

The global COVID-19 pandemic mobilized both the scientific community and the pharmaceutical industry, with the aim of designing vaccines and new antiviral drugs. Unfortunately, the drug discovery process is tedious and expensive. With introduction of machine learning algorithms and growth of computational power; high-throughput computing; and new, fast and reliable models with property predicting potentials became a pillar of the computer-aided drug design. This represents an opportunity to identify, develop and optimize potential drugs in a pace that was unimaginable even 10 years ago.

**Summary points**

**Results & discussion**

- Two SARS-CoV-2 3CLpro conformations are identified, differing in the relative position of the domain III.
- Geometry of the active side and the groove between domains II and III are conformation dependent.
- Results of the molecular docking experiments were used to rank microbial natural products as potential inhibitors of SARS-CoV-2 3CLpro.
- ADMET properties of microbial natural products were the final filter for selecting the top 8 hit molecules.

**Conclusion**

- A groove binding site is suggested as a potential target for prevention of enzyme maturation.
- Molecular dynamics simulations for the most potent natural product, futalosine, demonstrated the stability of the SARS-CoV-2 3CLpro–futalosine complexes.
