# Supplementary material for: Can natural products stop the SARS-CoV-2 virus? A docking and molecular dynamics study of a natural product database
Source: Future Med Chem. 2021 Jan 8:10.4155/fmc-2020-0248. doi: 10.4155/fmc-2020-0248 (PMC7798421; doi:10.4155/fmc-2020-0248)
Supplement: Supplementary file 1 [file Supplementary_data.docx]

**Can natural products stop the SARS-CoV-2 virus? A docking and molecular dynamics study of a natural product database**

Jurica Novak^1,*^, Hrvoje Rimac^1,2^, Shivananda Kandagalla^1^, Maria A. Grishina^1^, Vladimir A. Potemkin^1^

1 Laboratory of Computational Modeling of Drugs, Higher Medical and Biological School, South Ural State University, Chelyabinsk, 454008, Russia, Chaikovskogo 20A

2 Department of Medicinal Chemistry, University of Zagreb, Faculty of Pharmacy and Biochemistry, 10000 Zagreb, Croatia, Ante Kovacica 1

Corresponding author:

E-mail: novaki@susu.ru

SUPPORTING INFORMATION

Table SI 1. Clustering statistics for unbound SARS-CoV-2 3CLpro.

| # of clusters | DBI ^a^ | pSF ^b^ | SSR/SST ^c^ |
| --- | --- | --- | --- |
| 2 | 0.703 | 268465 | 0.417 |
| 3 | 1.674 | 192884 | 0.507 |
| 4 | 1.779 | 156434 | 0.556 |
| 5 | 1.825 | 131547 | 0.584 |

^a^ DBI = Davies-Bouldin index, ^b^ pSF = pseudo F statistic, ^c^ SSR/SST = ratio of sum of squares regression and sum of squares error

Table SI 2. Fraction of time that the unbound SARS-CoV-2 3CLpro spends in different conformations (fraction) and the average distance to centroid (r_AD2C_) for different numbers of clusters (k).

| # cluster | k = 2 | |
| --- | --- | --- |
|  | Fraction | r_AD2C_ / Å |
| 1 | 0.867 | 1.41 |
| 2 | 0.133 | 1.03 |
|  | k = 3 | |
| 1 | 0.539 | 1.25 |
| 2 | 0.329 | 1.35 |
| 3 | 0.132 | 1.02 |
|  | k = 4 | |
| 1 | 0.319 | 1.17 |
| 2 | 0.308 | 1.20 |
| 3 | 0.242 | 1.29 |
| 4 | 0.131 | 1.02 |
|  | k = 5 | |
| 1 | 0.309 | 1.17 |
| 2 | 0.268 | 1.17 |
| 3 | 0.157 | 1.18 |
| 4 | 0.134 | 1.19 |
| 5 | 0.131 | 1.02 |

Table SI 3. Predicted ADMET properties using pkCSM web server.^a^

| ATLAS ID (alternative name) | CYP1A2  inhibitor | CYP2C9 inhibitor | CYP2D6 inhibitor | CYP3A4 inhibitor | P-glycoprotein I/II inhibitor | hERG I/II inhibitor | AMES | logP | # HB acceptor sites | # HB donor sites |
| --- | --- | --- | --- | --- | --- | --- | --- | --- | --- | --- |
| NPA013652 (19-hydroxypenitrem A) | No | No | No | No | No | No | Negative | 4.7766 | 7 | 5 |
| NPA001702 (2',3'-Epoxymyrothecine A) | No | No | No | No | No | No | Negative | 0.6636 | 10 | 3 |
| NPA002809 (Futalosine) | No | No | No | No | No | No | Negative | 0.1001 | 9 | 4 |
| NPA005589 (Pseudonocardone C) | No | No | No | No | No | No | Negative | 0.9088 | 10 | 5 |
| NPA022742 (MDN-0185) | No | No | No | No | No | No | Negative | 0.9639 | 10 | 4 |
| NPA013618 (Pityriarubin A) | No | No | No | No | No | No | Negative | 4.6875 | 4 | 5 |
| NPA015941 (Izumiphenazine B) | No | No | No | No | No | No | Negative | 3.4539 | 9 | 4 |
| NPA010921 (Verrucarin Y) | No | No | No | No | No | No | negative | 2.1846 | 9 | 1 |

^a^ Pires DEV, Blundell TL, Ascher DB. pkCSM: Predicting small-molecule pharmacokinetic and toxicity properties using graph-based signatures. *J. Med. Chem.* 58(9), 4066–4072 (2015).

# HB = number of hydrogen bond


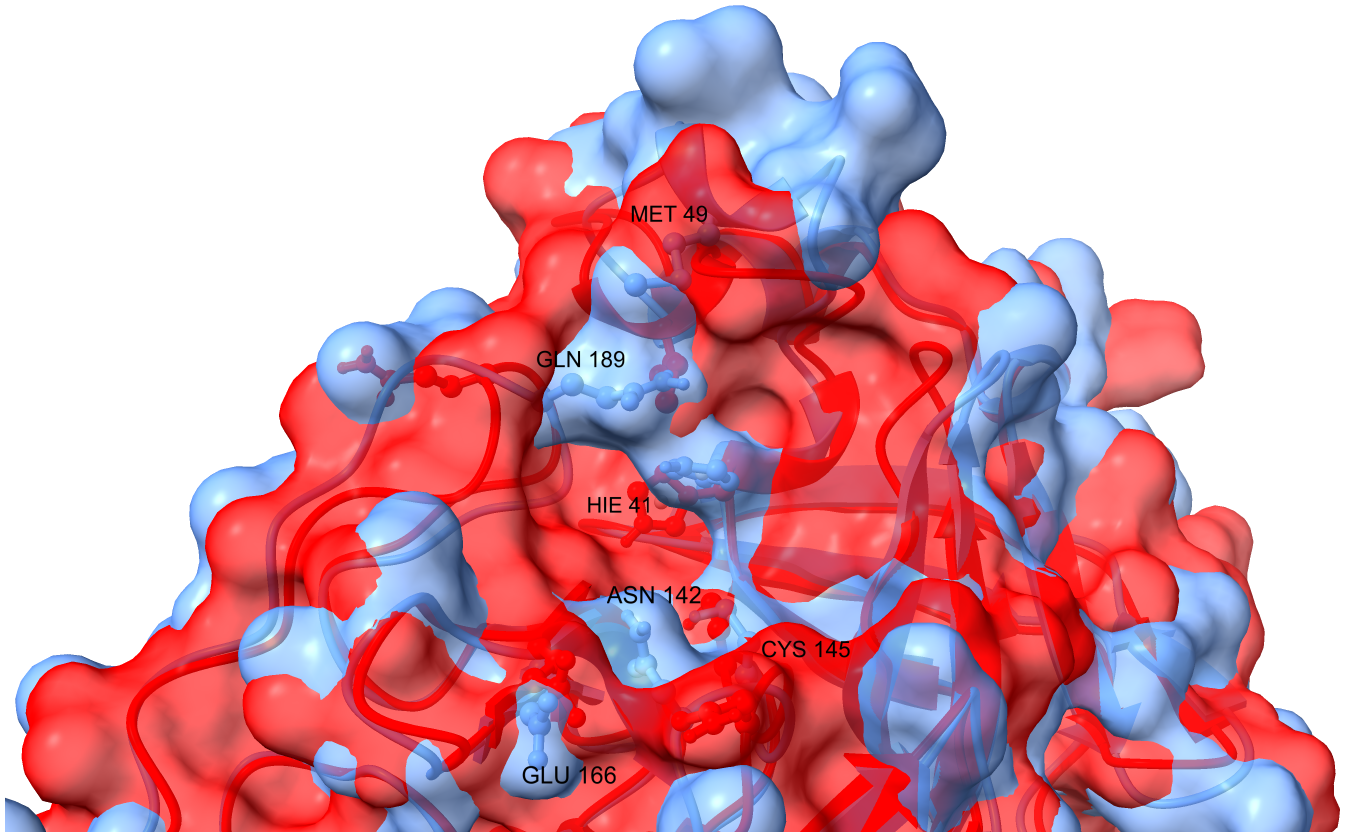


Figure SI 1. Overlaid structures of the representative primary (red) and the secondary (blue) conformations of the SARS-CoV-2 3CLpro extracted from the MD simulation, highlighting the changes in the catalytic pocket between the two conformations.


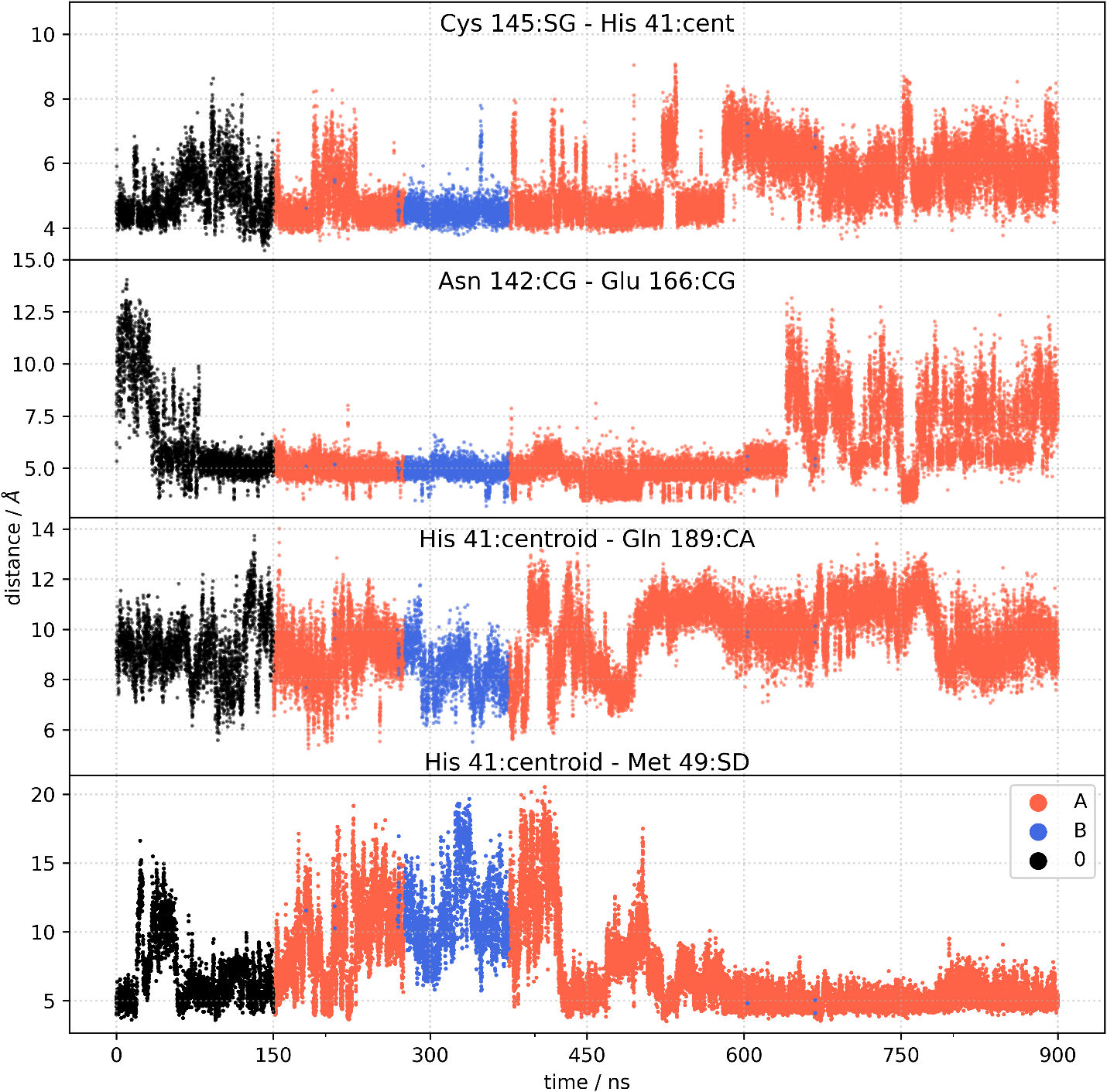


Figure SI 2. Evolution of selected distances of the free SARS-CoV-2 3CLpro with clusters shown. Conformation **A** (A), conformation **B** (B), equilibration phase (0).

| 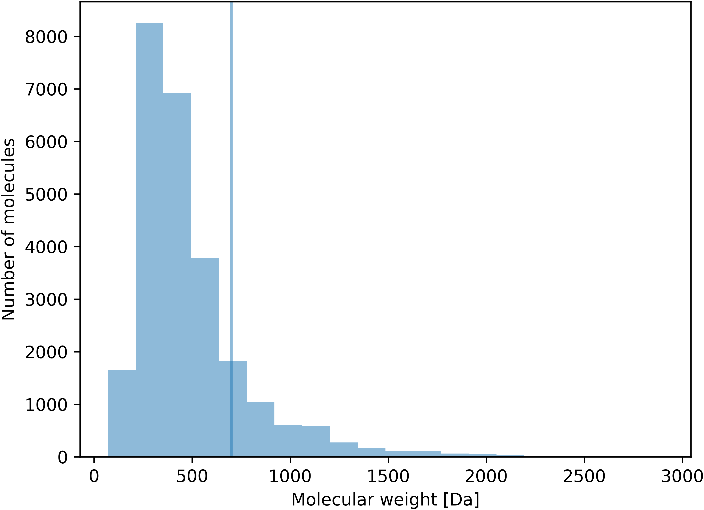 | 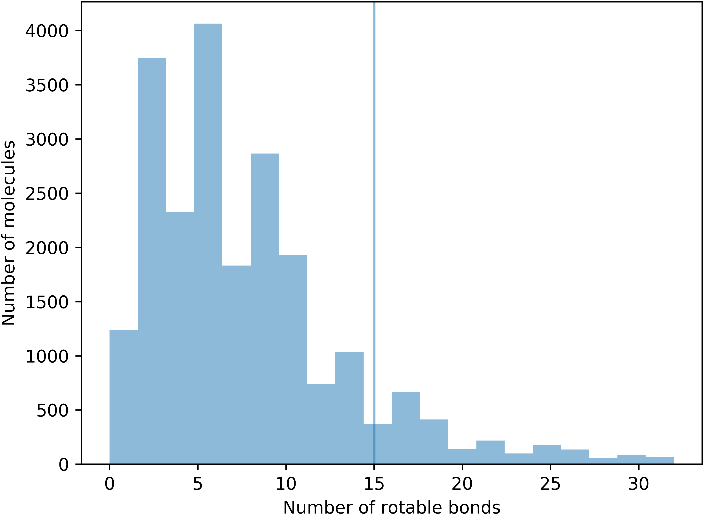 |
| --- | --- |

Figure SI 3. The Natural Products Atlas database distribution of molecular weights (left) and distribution of binding energies as a result of virtual screening to SARS-CoV-2 3CLpro (right) (PDB entry 6LU7).

Table SI 4. Geometrical parameters of the catalytic pock24-27et^a^ of the SARS-CoV-2 3CLpro for crystal structure (PDB ID 6LU7) and two representative conformations from MD simulation.

|  | 6LU7 | **A** | **B** |
| --- | --- | --- | --- |
| RMSD^b^ from 6LU7 / Å | - | 2.628 | 2.408 |
| d (145@SG:41@cent) | 4.382 | 4.744 | 5.667 |
| d (142@CG:166@CG) | 9.886 | 7.219 | 4.708 |
| d (41@cent:189@CA) | 9.858 | 8.864 | 7.149 |
| d (41@cent:41@SD) | 6.802 | 5.832 | 8.204 |
| SAS^c, e^ / Å^2^ | 351.125 | 389.829 | 355.580 |
| SAV^d, e^ / Å^3^ | 319.370 | 222.429 | 199.295 |

^a^ The catalytic pocket is defined as the space enclosed by amino acid residues within 5.5 Å of Cys 145, and includes residues with indices 24-27, 41, 49, 54, 140-145, 163-168, 172 and 187-192. ^b^ RMSD is calculated for the backbone atoms of the catalytic pocket residues. ^c^ SAS – solvent accessible surface. ^d^ SAV – solvent accessible volume. ^e^ SAS and SAV are calculated according to [1], with the default probe size of 1.4 Å.


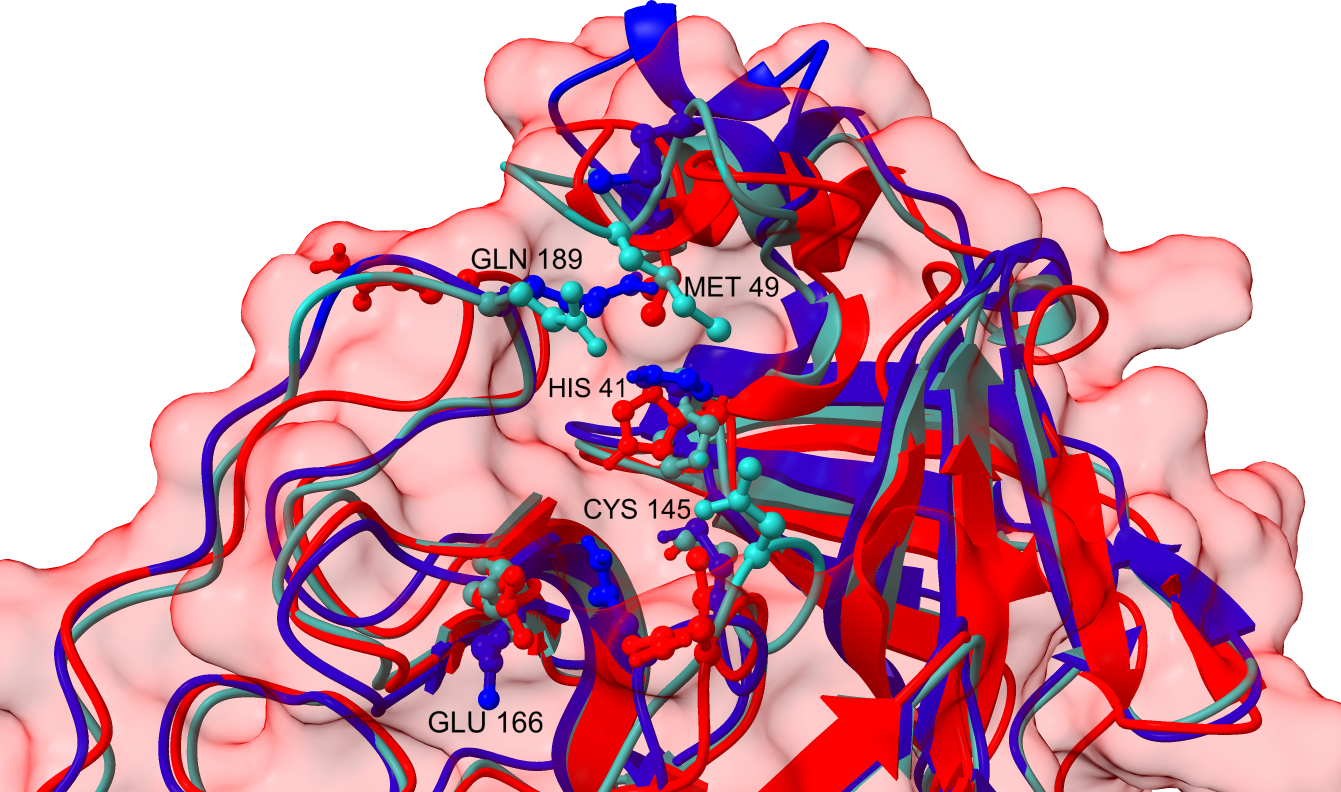


Figure SI 4. Overlaid structures of the representative primary (red) and secondary (blue) conformations and the crystal structure (light sea green) of the SARS-CoV-2 3CLpro highlighting changes of the geometry of the catalytic pocket.

| 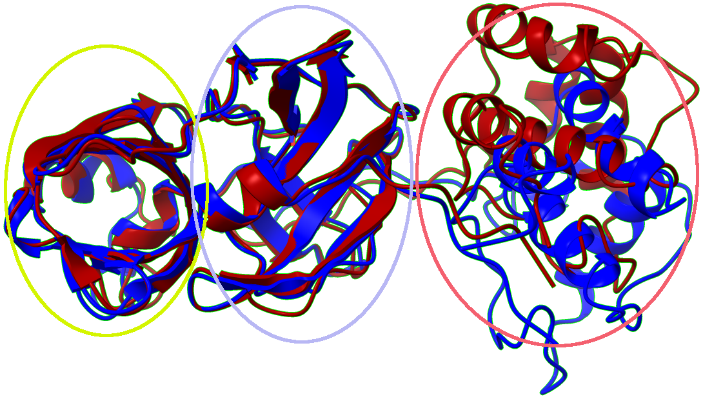 | 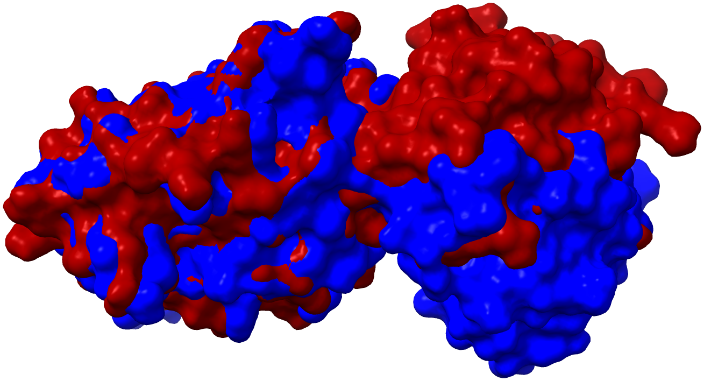 |
| --- | --- |

Figure SI 5. Overlaid SARS-CoV-2 3CLpro **A** and **B** conformations, highlighting the changes in the domain III region (left) and in the groove between domains II and III (right).

Table SI 5. Top three DrugBank molecules and their Tanimoto molecular similarity index (T_c_) for the top hit molecules.

| Hit molecule | DrugBank 1 | DrugBank 2 | DrugBank 3 |
| --- | --- | --- | --- |
| 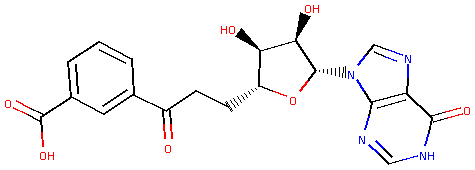 | 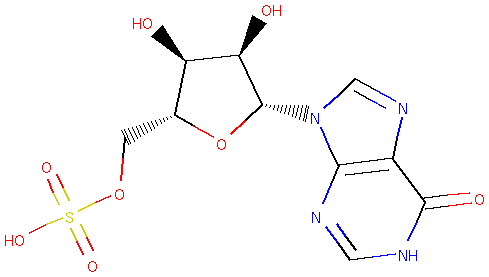 | 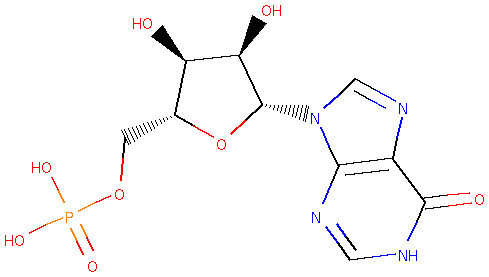 | 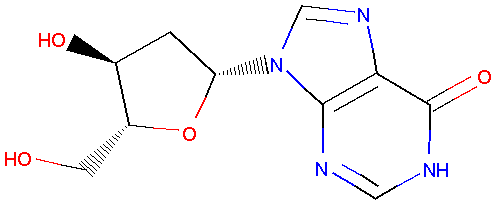 |
| NPA002809  (Futalosine) | DB05973  T_c_ = 0.489 | DB04566  T_c_ = 0.479 | DB02380  T_c_ = 0.394 |
| 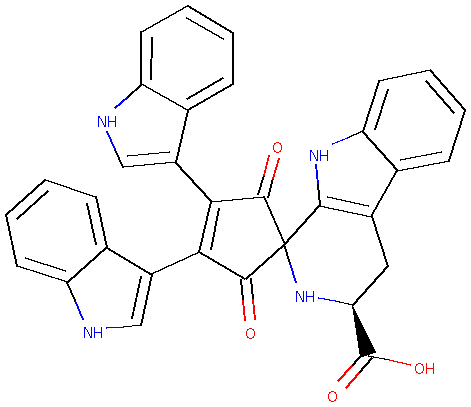 | 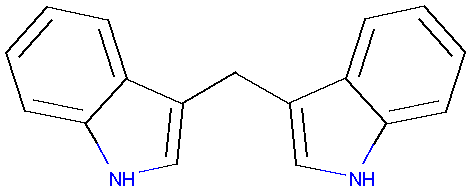 | 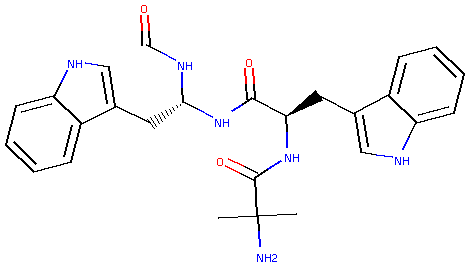 | 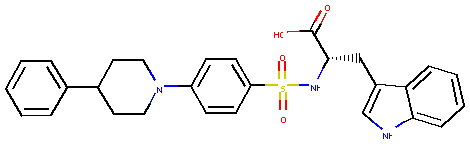 |
| NPA013618  (Pityriarubin A) | DB11875  T_c_ = 0.346 | DB13074  T_c_ = 0.343 | DB02449  T_c_ = 0.312 |
| 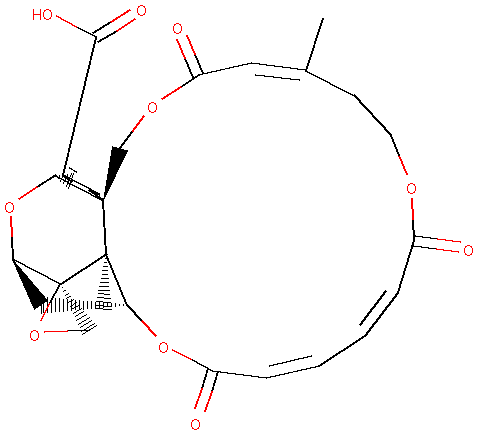 | 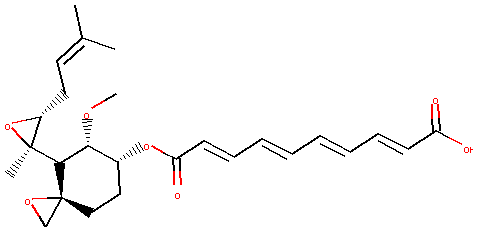 | 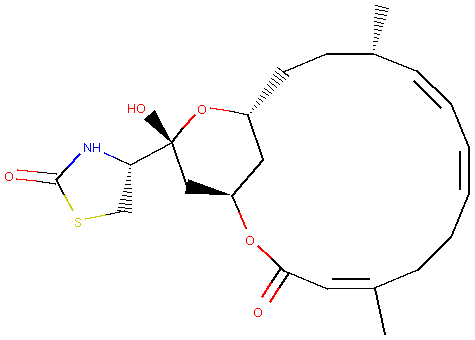 | 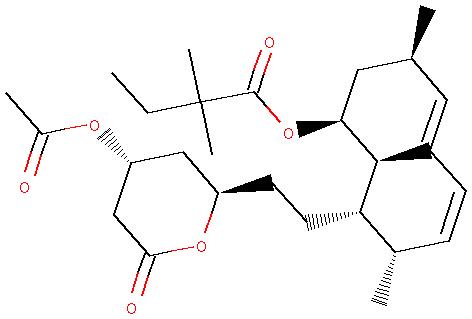 |
| NPA010921  (Verrucarin Y) | DB02640  T_c_ = 0.326 | DB02621  T_c_ = 0.257 | DB14648  T_c_ = 0.253 |
| 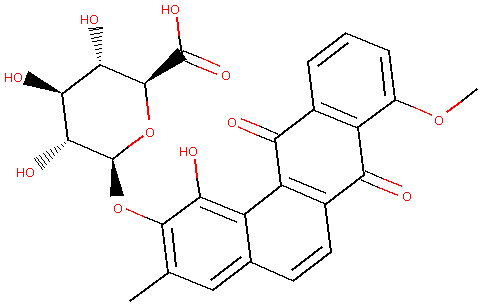 | 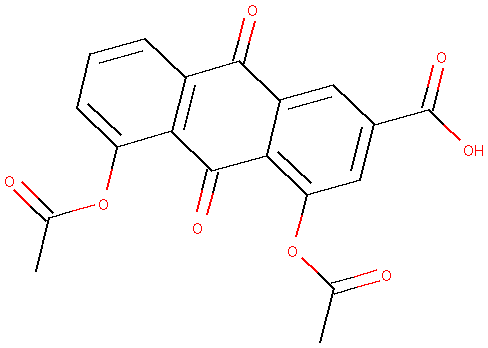 | 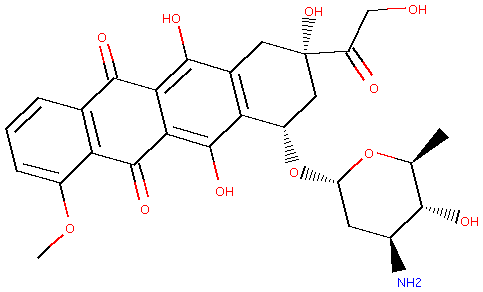 | 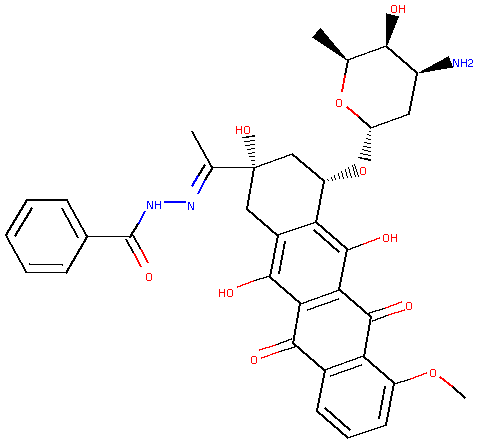 |
| NPA005589  (Pseudonocardone C) | DB11994  T_c_ = 0.319 | DB00445  T_c_ = 0.308 | DB11618  T_c_ = 0.306 |
| 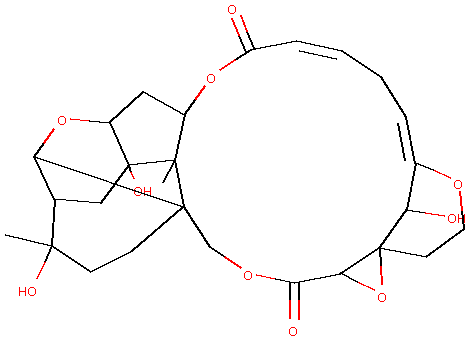 | 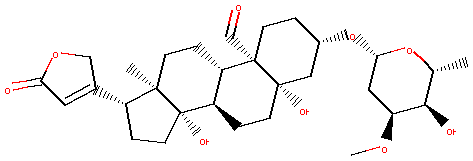 | 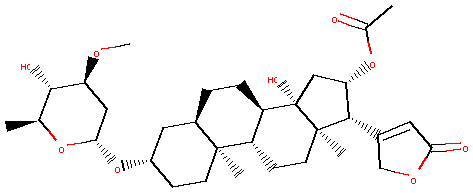 | 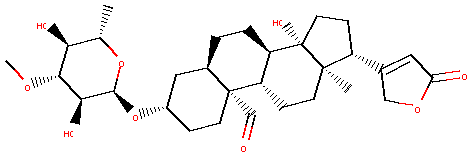 |
| NPA001702  (2',3'-Epoxymyrothecine A) | DB13240  T_c_ = 0.277 | DB12843  T_c_ = 0.276 | DB13756  T_c_ = 0.272 |
| 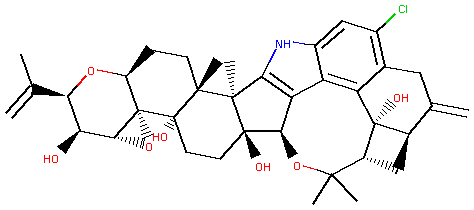 | 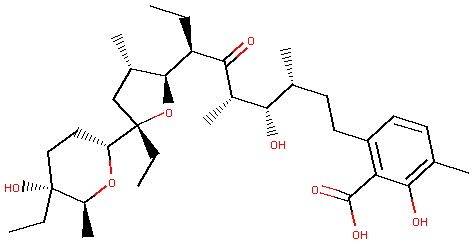 | 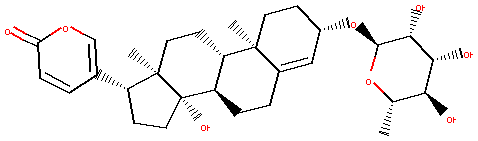 | 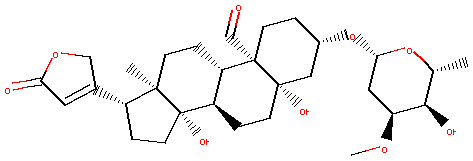 |
| NPA013652  (19-hydroxypenitrem A) | DB11423  T_c_ = 0.256 | DB13307  T_c_ = 0.227 | DB13240  T_c_ = 0.221 |
| 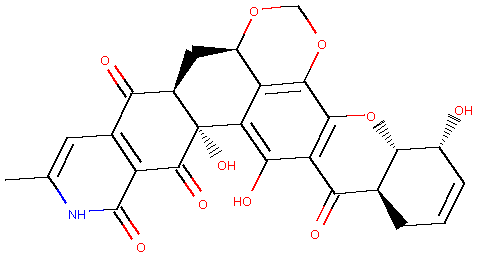 | 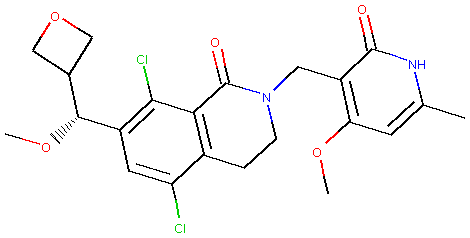 | 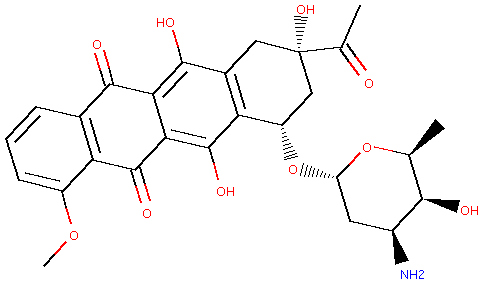 | 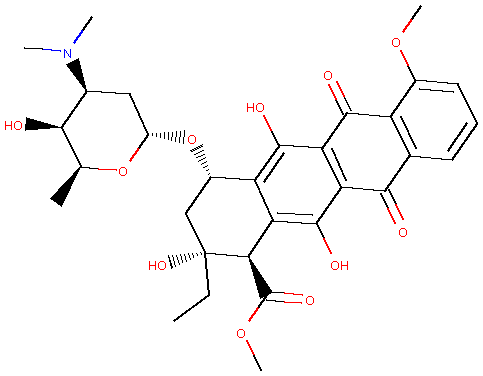 |
| NPA022742  (MDN-0185) | DB14799  T_c_ = 0.242 | DB00694  T_c_ = 0.240 | DB03199  T_c_ = 0.230 |
| 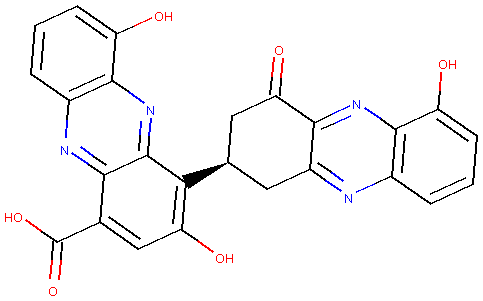 | 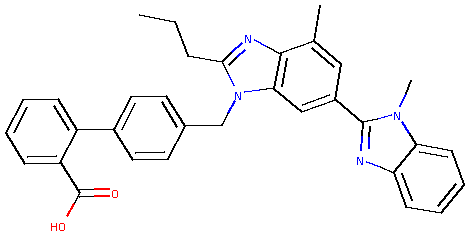 | 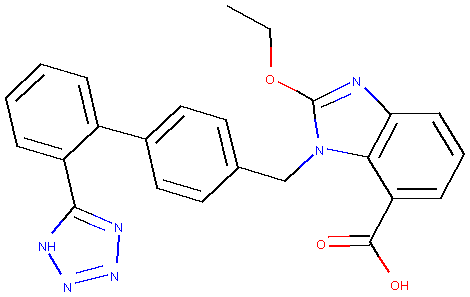 | 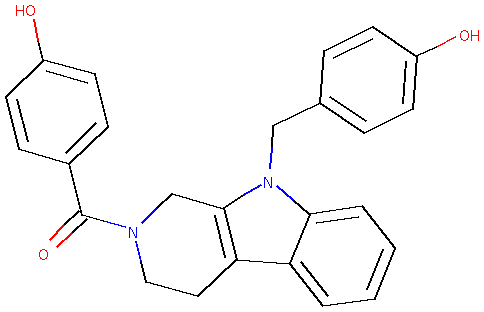 |
| NPA015941  (Izumiphenazine B) | DB00966  T_c_ = 0.237 | DB13919  T_c_ = 0.230 | DB04030  T_c_ = 0.229 |


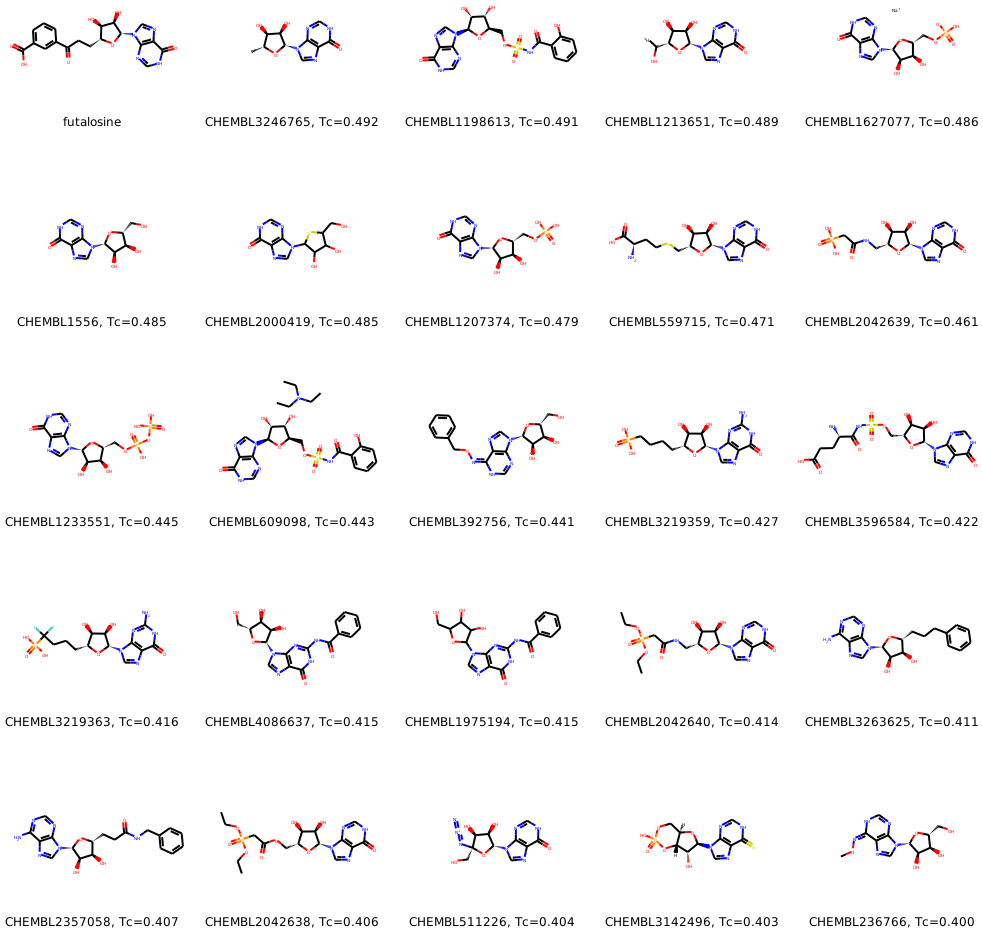


Figure SI 6. Top 25 molecules from the ChEMBL database with the highest Tanimoto molecular similarity index (T_c_) compared to futalosine.


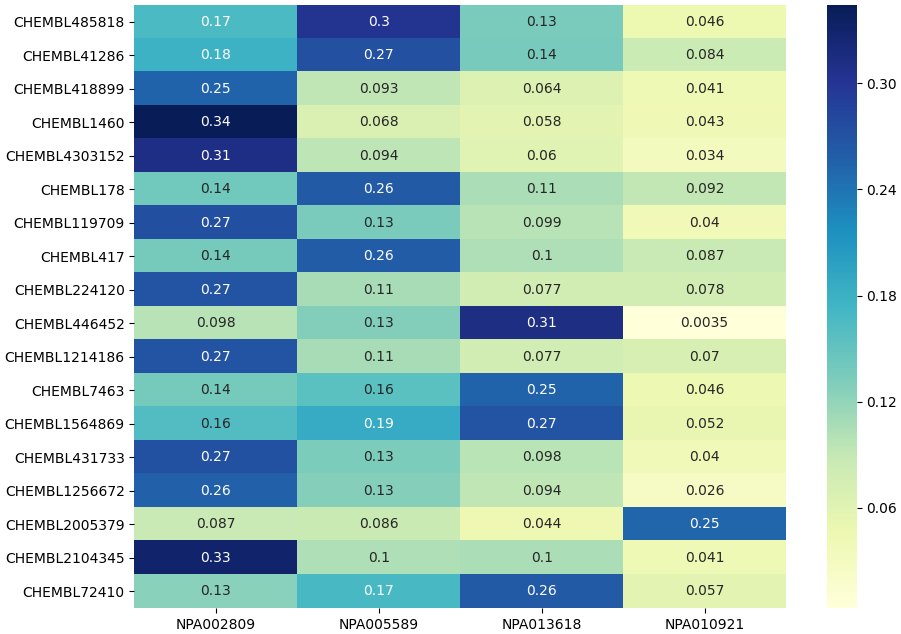


Figure SI 7. Heat map presenting molecular similarity of the top hit molecules (with the threshold value of maximal Tanimoto index for hit molecules being 0.25) from Natural Products Atlas against 5543 ChEMBEL compounds tested for its activity against SARS-CoV-2.


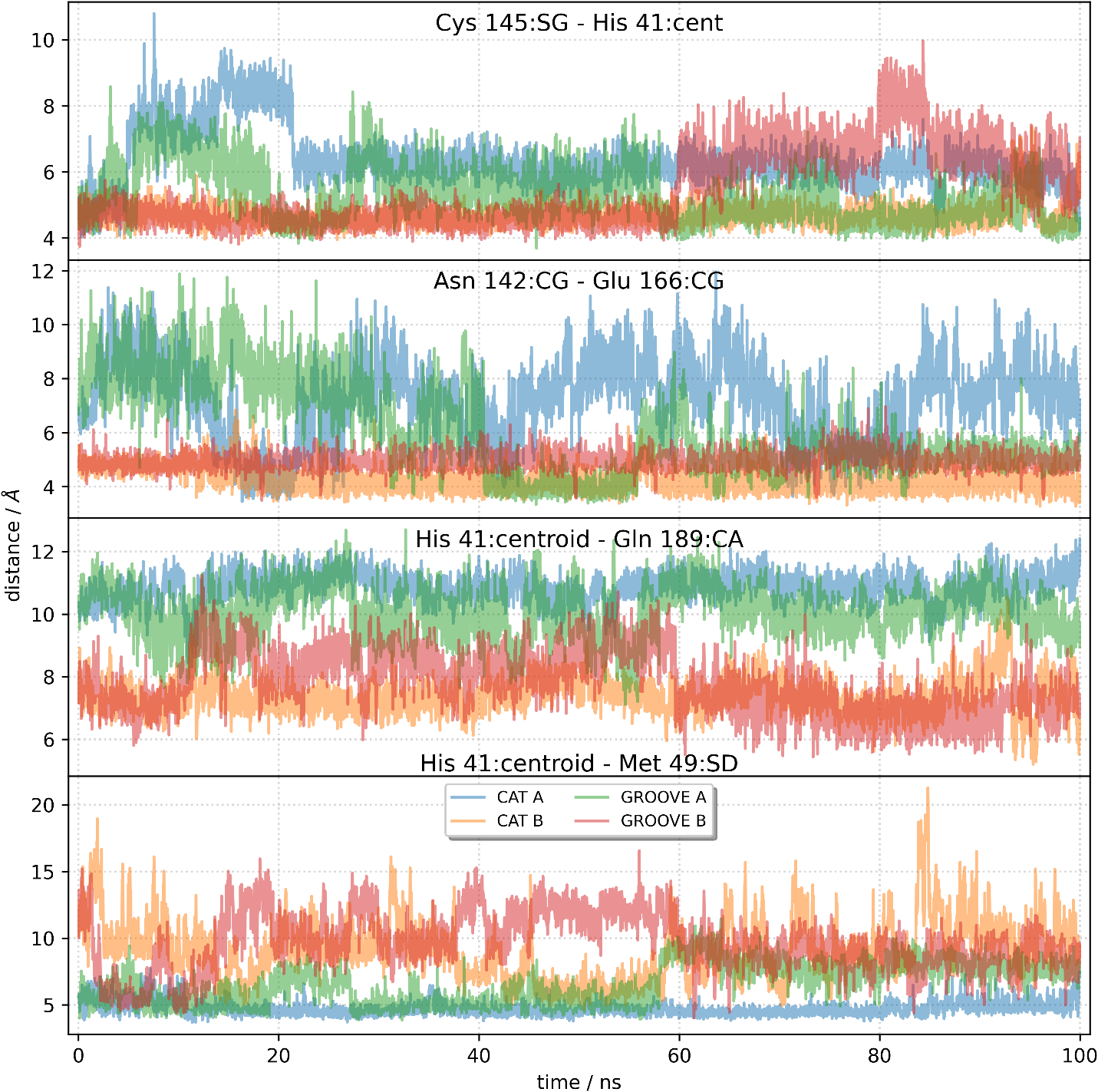


Figure SI 8. Evolution of selected distances of the SARS-CoV-2 3CLpro with futalosine bound in the catalytic (CAT) or in the groove (GROOVE) site, for conformations **A** and **B** during 100 ns simulation time.

*
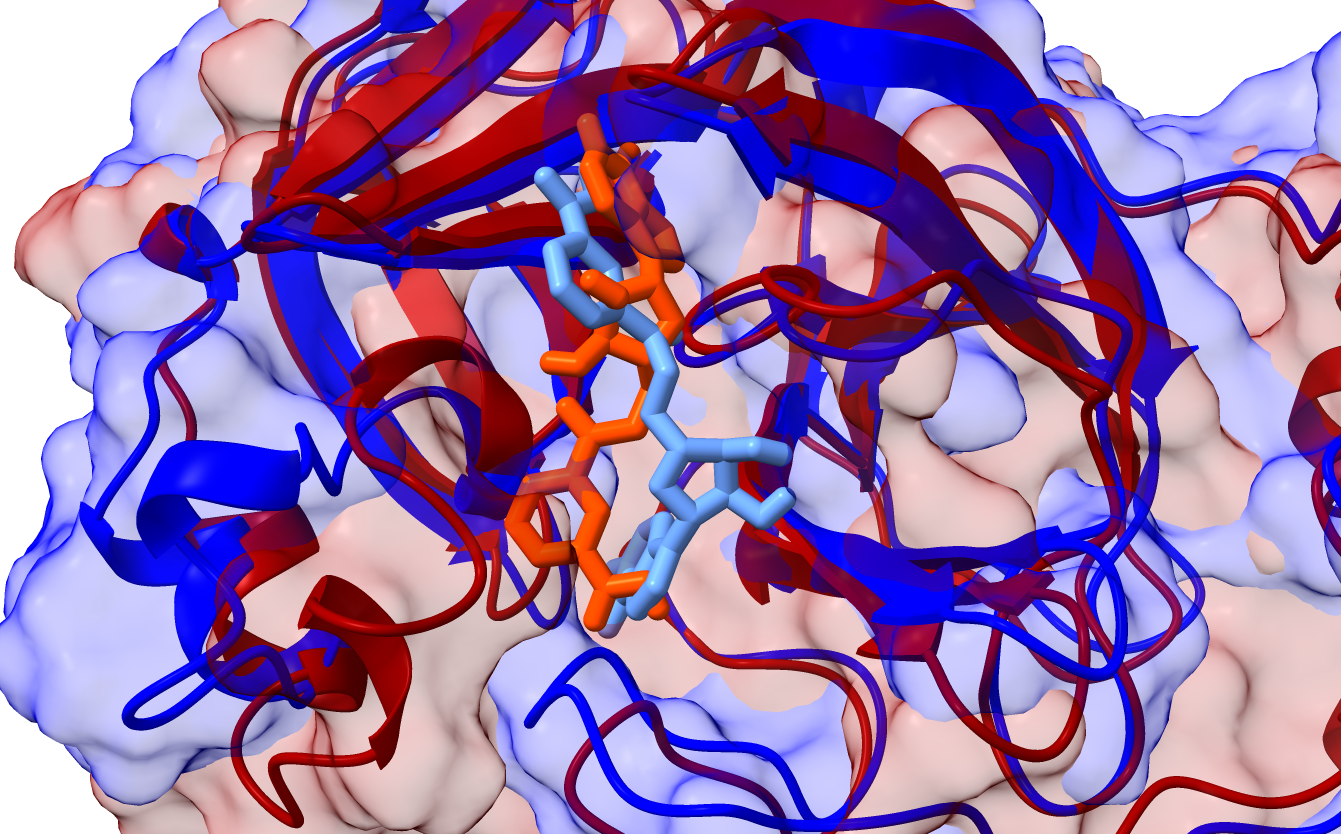
*

Figure SI 9. A comparison of the SARS-CoV-2 3CLpro : futalosine complexes at the end of 100 ns molecular dynamics. Conformations **A** (red) and **B** (blue).

REFERENCES:

1. Tian W, Chen C, Lei X, Zhao J, Liang J. CASTp 3.0: Computed atlas of surface topography of proteins. *Nucleic Acids Res.* 46(W1), W363–W367 (2018).
